# T cell costimulation blockade blunts pressure overload-induced heart failure

**DOI:** 10.1038/ncomms14680

**Published:** 2017-03-06

**Authors:** Marinos Kallikourdis, Elisa Martini, Pierluigi Carullo, Claudia Sardi, Giuliana Roselli, Carolina M. Greco, Debora Vignali, Federica Riva, Anne Marie Ormbostad Berre, Tomas O. Stølen, Andrea Fumero, Giuseppe Faggian, Elisa Di Pasquale, Leonardo Elia, Cristiano Rumio, Daniele Catalucci, Roberto Papait, Gianluigi Condorelli

**Affiliations:** 1 Adaptive Immunity Laboratory, Humanitas Clinical and Research Center, Via Manzoni 56, Rozzano, 20089 Milan, Italy; 2 Department of Biomedical Sciences, Humanitas University, Via Manzoni 113, Rozzano, 20089 Milan, Italy; 3 Department of Cardiovascular Medicine, Humanitas Clinical and Research Center, Via Manzoni 56, Rozzano, 20089 Milan, Italy; 4 Institute of Genetic and Biomedical Research (IRGB)—UOS of Milan, National Research Council of Italy, Via Manzoni 56, Rozzano, 20089 Milan, Italy; 5 Department of Veterinary Medicine (DIMEVET), Università degli Studi di Milano, Via Celoria 10, 20133 Milan, Italy; 6 KG Jebsen Centre of Medicine, Department of Circulation and Medical Imaging, Norwegian University of Science and Technology, Postboks 8905, 7491 Trondheim, Norway; 7 Norwegian Health Association, Oscars gate 36A, 0258 Oslo, Norway; 8 Cardiac Surgery, Humanitas Clinical and Research Center, Via Manzoni 56, Rozzano, 20089 Milan, Italy; 9 Department of Cardiac Surgery, University of Verona, 37129 Verona, Italy; 10 Department of Molecular and Translational Medicine, University of Brescia, 25123 Brescia, Italy; 11 Dipartimento di Scienze Farmacologiche e Biomolecolari, Università degli Studi di Milano, Via Trentacoste 2, 20133 Milan, Italy; 12 Laboratory of Signal Transduction in Cardiac Pathologies, Humanitas Clinical and Research Center, Via Manzoni 56, Rozzano, 20089 Milan, Italy

## Abstract

Heart failure (HF) is a leading cause of mortality. Inflammation is implicated in HF, yet clinical trials targeting pro-inflammatory cytokines in HF were unsuccessful, possibly due to redundant functions of individual cytokines. Searching for better cardiac inflammation targets, here we link T cells with HF development in a mouse model of pathological cardiac hypertrophy and in human HF patients. T cell costimulation blockade, through FDA-approved rheumatoid arthritis drug abatacept, leads to highly significant delay in progression and decreased severity of cardiac dysfunction in the mouse HF model. The therapeutic effect occurs via inhibition of activation and cardiac infiltration of T cells and macrophages, leading to reduced cardiomyocyte death. Abatacept treatment also induces production of anti-inflammatory cytokine interleukin-10 (IL-10). IL-10-deficient mice are refractive to treatment, while protection could be rescued by transfer of IL-10-sufficient B cells. These results suggest that T cell costimulation blockade might be therapeutically exploited to treat HF.

## Abstract (web summary)

Abatacept is an FDA-approved drug used for treatment of rheumatoid arthritis. Here the authors show that abatacept reduces cardiomyocyte death in a mouse model of heart failure by inhibiting activation and heart infiltration of T cells and macrophages, an effect mediated by IL-10, suggesting a potential therapy for heart failure.

Heart failure (HF) is a major cause of hospitalization, morbidity and mortality; it is often encountered as the final stage of pathological cardiac hypertrophy and fibrosis brought about by hemodynamic overload^1^. Some forms of cardiomyopathy—termed inflammatory cardiomyopathies—are caused by autoimmunity or by immune responses to infection, indicating that cardiac dysfunction can also result from disease of the immune system^2^. Intriguingly, recent studies have uncovered that HF induced by hemodynamic overload also involves a significant inflammatory component^3^,^4^,^5^. This inflammation is characterized by the presence of innate immune cells (macrophages) in the myocardium and upregulation of pro-inflammatory cytokines, such as tumour-necrosis factor-α, interleukin (IL)-6 and IL-1β, which impact negatively on disease outcome^3^,^6^,^7^. Even though its absence can be compensated^8^, IL-6 administration is sufficient to set off the process leading to pathological cardiac hypertrophy^9^. Innate immune cells and cytokines are believed to promote cardiac inflammation, worsening disease outcome.

Although the concept of inflammation as a major component of HF is consolidated^10^, clinical trials attempting to combat HF by blocking cytokines have not been successful^5^,^11^. The reason for this failure could be the redundant function of individual cytokines^8^. Therefore, in order to identify more suitable immunotherapy targets for HF, we need to better characterize the involvement and hierarchy of different soluble and cellular (innate and adaptive) immune mediators in the disease.

The innate immune system acts as a non-specific, but effective and rapid, first line of defense against pathogens. During long-lasting responses, however, it becomes subject to the control of the adaptive immune system's T lymphocytes (T cells)^12^, which, along with B cells, mediate antigen-specific immune responses. Therefore, T cells, if involved in HF pathogenesis, could become attractive and more specific immunotargets for therapeutic intervention. This assumption is supported by the implication of T cells in pressure overload-induced cardiac fibrosis^13^.

Here we identified the immune mediators involved in pressure overload-induced HF, finding that T cells infiltrated the pathologically hypertrophic myocardium, in line with their role in long-lasting inflammation. Indeed, inflammation was a key factor distinguishing pathological hypertrophy from physiological, ‘benign' hypertrophy, which occurs during exercise training. Taking advantage of the presence of T cells, we utilized abatacept—an Food and Drug Administration (FDA)-approved CTLA4-Ig fusion protein that blocks T cell costimulation, selectively inhibiting pro-inflammatory T cell function^14^—to significantly blunt cardiac dysfunction in a mouse HF model. Inhibition of disease progression was achieved even when the drug was administered at an advanced stage of the pathology. Abatacept systemically inhibited T cell activation, cardiac macrophage maturation and reduced cardiac T cell and macrophage infiltration, leading to reduced cardiomyocyte death. The protective effect was lost in the absence of anti-inflammatory cytokine interleukin-10 (IL-10), which was produced mostly by B cells. Adoptive transfer of IL-10-sufficient B cells but not T cells into IL-10-deficient recipient mice in the HF model rescued the loss of protection. Taken together, our findings indicate that T cell-mediated responses are involved in the development of pathological cardiac hypertrophy and that interfering with these responses, using existing, clinically validated strategies, has the potential to become a therapeutic option for HF.

## Results

### Analysis of immune mediators during the progression to HF

**Figure 1:**
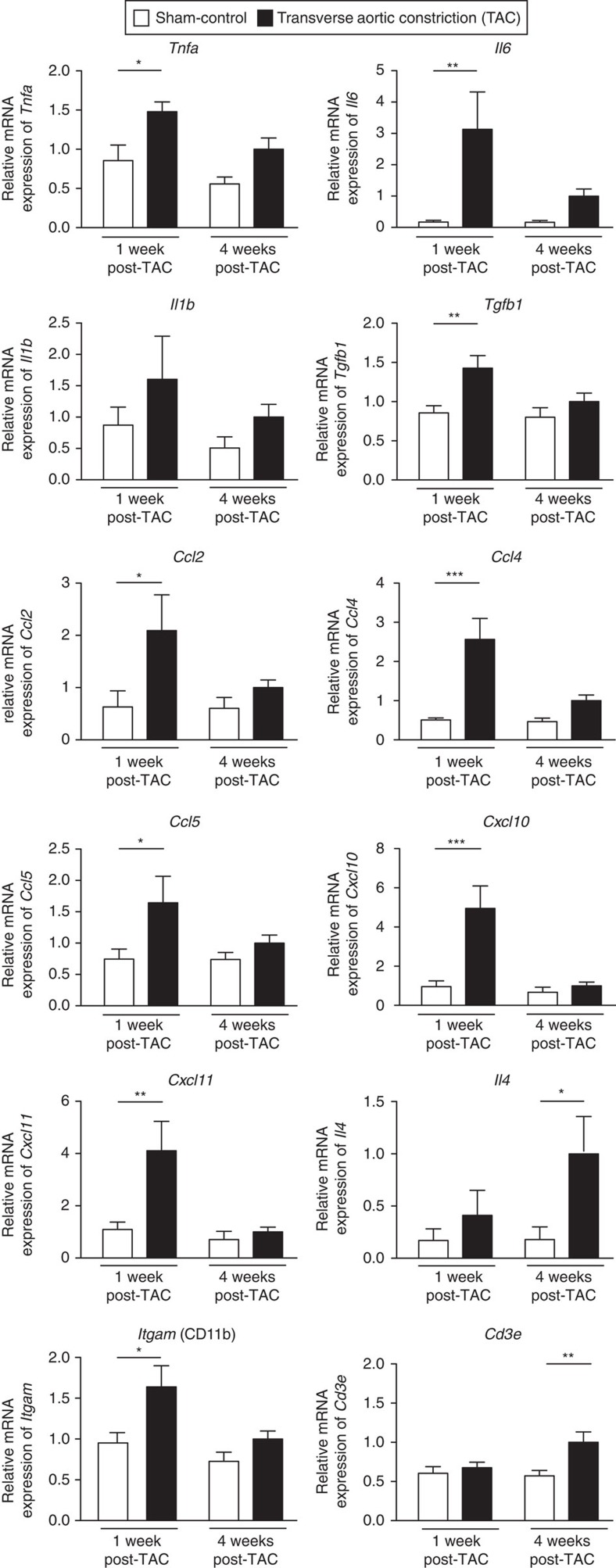
The inflammatory signature in hypertrophic left ventricle of mice. Gene expression analysis (TaqMan real-time qPCR) of mediators of inflammation within the left ventricle of C57BL6/J mice. Relative mRNA expression in sham-operated control mice (white bars) and TAC-operated mice (black bars) at 1 and 4 weeks after surgery, internally normalized to 18 s ribosomal RNA expression. *Tnfa*, *Il6*, *Tgfb1*, *Ccl2*, *Ccl4*, *Ccl5*, *Cxcl10*, *Cxcl11* and the innate cell marker *Itgam* (CD11b) were significantly increased in the TAC group compared with sham, 1 week after TAC. Four weeks after the operation, *Il4* and the T cell marker *Cd3e* were significantly increased. Values are mean±s.e.m. (*n*=7–9). Two-way analysis of variance (ANOVA), Bonferroni post-test: *P value<0.05; **P value<0.01; ***P value<0.001.

We subjected mice to transverse aortic constriction (TAC), the standard model for pathological cardiac hypertrophy^15^, and assessed the presence of soluble and cellular immune mediators within the myocardium via quantitative PCR (qPCR) at 1 and 4 weeks after TAC surgery (Fig. 1). Cardiac functionality was monitored via regular transthoracic echocardiography (Supplementary Table 1). At 1 week post-TAC, we found a significant upregulation of *Tnfa* and *Il6*, as previously described^7^,^16^. Cells of the immune system are recruited to and/or retained at their sites of action via chemokines. We found a significant early expression of *Ccl2* and C*xcl11* (ref. 17) as well as C*cl4*, C*cl5* and C*xcl10* (Fig. 1), the majority of which are markers of a type 1 (M1/Th1)-polarized inflammatory response^18^. *Itgam* (CD11b), a hallmark of the presence of innate immune cells, such as macrophages or monocytes, was also upregulated 1 week post-TAC, suggesting that type 1-polarized innate immune cells are recruited to the stressed myocardium early on.

We observed significant upregulation of the T-cell-specific marker *Cd3e* at 4 weeks post-operation, suggesting that T cells expand or are recruited to the stressed left ventricle at this later timepoint. Concurrent upregulation of *Il4*, a hallmark of type 2 (M2/Th2)-polarized responses, is compatible with a gradual shift from an M1 to an M2/Th2 response as the myocardium progresses toward HF, though this is speculative. Th2-polarized T cells promote fibrosis in other pathological conditions^19^. Transcripts of cytokines that characterize Th1 and Th17 responses, such as *Ifng* and *Il17*, or of the anti-inflammatory cytokine *Il10* were not significantly altered (Supplementary Fig. 1a).

We asked whether the onset of inflammation correlated with T cell infiltration and/or proliferation. Assuming a linear regression model, we first examined the correlation between *Cd3e* expression (indicative of T cell presence) and *Il6* expression (indicative of inflammation) in samples derived from TAC-operated mice, 4 weeks post-operation. The results (Supplementary Fig. 1b, red line) show a significant positive slope, suggesting that such a correlation exists. A likely interpretation would be that inflammation drives the infiltration and/or proliferation of T cells into the myocardium. Repeating the analysis for sham-operated animals (Supplementary Fig. 1b, blue line) also yielded a significant positive slope, however with lower mean *il6* and *cd3e* values. This suggests that, even in the absence of the aortic constriction, the limited (but nonetheless present) inflammation generated by the sham operation (which does involve surgery, albeit without permanent constriction) may be leading to a limited infiltration/proliferation of T cells, even if this is significantly lower than in TAC (as shown in Fig. 1).

### Immune response mediator absence in physiological hypertrophy

The above show that pathological cardiac hypertrophy, which leads to fibrosis and HF, is associated with inflammation. Yet non-pathological forms of cardiac hypertrophy also exist. The most physiologically relevant model for these is exercise training. Mice subjected to a running program show ‘physiological' hypertrophy in which the increase in cardiomyocyte size is accompanied by an increased functionality of the cells and absence of fibrosis^20^,^21^. We thus asked whether the immune mediators that we identified in the TAC model of HF were also present in exercise-trained mice. We found no significant upregulation of immune response mediator transcripts in these mice (Supplementary Fig. 2a). This finding strongly suggests that, unlike pathological hypertrophy, physiological hypertrophy features a complete absence not only of fibrosis, but also of an innate and adaptive immune response. A more ‘artificial', non-pathological hypertrophy model, induced by cardiac-specific overexpression of the constitutively active E40K mutant of the serine-threonine kinase Akt in the heart^22^, displayed an incomplete array of pro-inflammatory mediators present in the left ventricle of 8-week-old Akt transgenic mice (Supplementary Fig. 2b). Altogether, these results support a positive association between inflammation and the pathological nature of cardiac hypertrophy.

### T cell presence in the stressed myocardium in mice and humans

**Figure 2:**
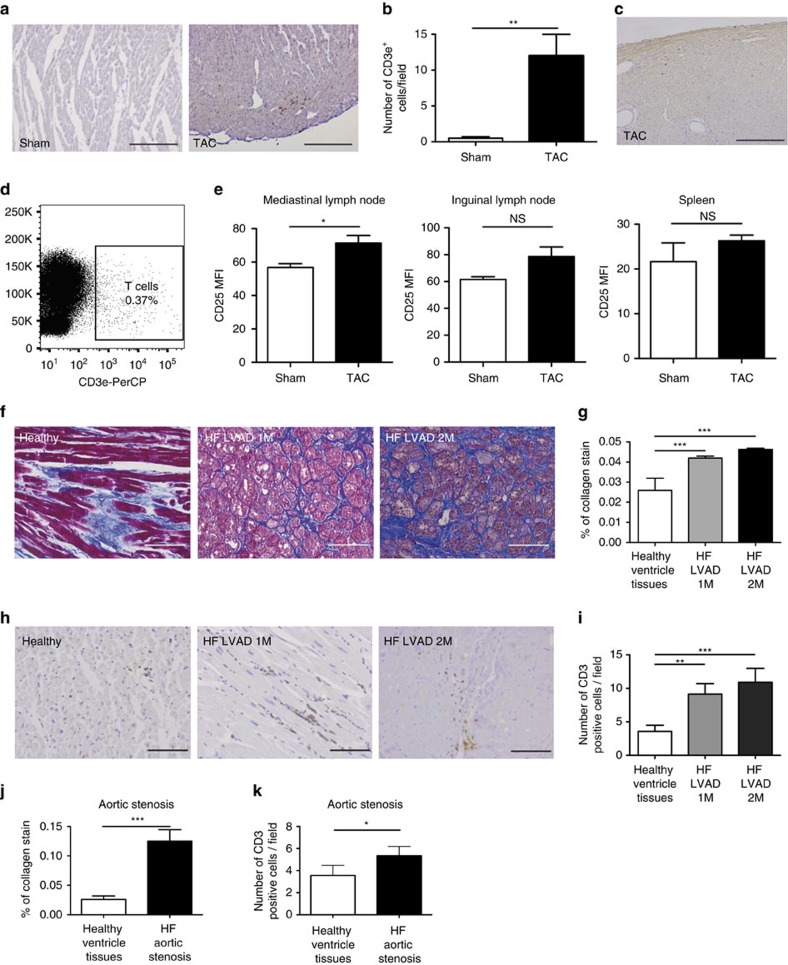
T cells in the ailing left ventricle. (**a**) Representative immunohistochemical (IHC) staining of left ventricles for CD3e (brown) in sham/TAC mice at 4 weeks. Original magnification 10 × ; bars=200 μm. (**b**) Summary of CD3e IHC. Mean±s.e.m. (*n*=6). Unpaired *t*-test. (**c**) Staining for CD3e (brown) in TAC-operated mice, 1 week post-operation. Original magnification 10 × ; bar=200 μm. (**d**) Representative fluorescence-activated cell sorting (FACS) analysis of CD3e^+^ cells from cardiac single cell suspension of TAC-operated mice 1 week post-operation. (**e**) FACS analysis of mediastinal (heart-draining) lymph nodes, inguinal lymph nodes and spleens 2 days post-operation. Mean fluorescence intensities of CD25 on CD3e^+^ cells. Mean±s.e.m.; sham (white bars), TAC (black bars) (*n*=4). Unpaired *t*-test. (**f**) Representative Azan's trichrome collagen staining (blue) of cardiac biopsies from healthy ventricle tissues (*n*=3), patients with severe dilated cardiomyopathy (DCM) due to mutation in lamin A/C, before placement of a left ventricular assist device (HF LVAD 1M) (*n*=4), and patients with more severe DCM due to mutation in lamin A/C and mutation in titin, before placement of a LVAD (HF LVAD 2M) (*n*=2) patients. Original magnification, 20 × ; bar=100 μm. (**g**) Statistical analysis of collagen deposition in ten identical regions of interest (ROIs), applied to all samples. Mean±s.e.m. Fisher's exact test for presence versus absence of fibrosis. Amount of collagen was also positively associated with disease severity (one-way analysis of variance (ANOVA); post-test for linear trend: *P*<0.001). (**h**) Representative staining for CD3e (brown) on the same samples as **f**. Bar=100 μm. (**i**) Statistical analysis of CD3e IHC analysis. Mean±s.e.m. One-way ANOVA with Dunn's post-test. (**j**) Statistical analysis of collagen deposition in cardiac biopsies from healthy ventricle tissues (*n*=3) and patients with HF from aortic stenosis (*n*=2) stained as in **f**. Mean±s.e.m. Healthy tissues (white bar), HF (black bars). Fisher's exact test for presence versus absence of fibrosis. (**k**) Statistical analysis of CD3e IHC analysis on the same samples as **j**. Healthy tissues (white bar), HF (black bars). Values are mean±s.e.m. Mann–Whitney test. For all tests *P value<0.05; **P value<0.01; ***P value<0.001.

Inhibition of inflammation as a strategy against HF has been attempted before, but the targets utilized resulted to be inadequate for this end^5^,^11^. T cells are required for the maintenance of long-term immune responses^12^ and thus could represent a better therapeutic target. Driven by the finding of T cell-specific C*d3e* messenger RNA (mRNA) upregulation in TAC mice at 4 weeks post-TAC, we further investigated the presence of T cells in pathological hypertrophy. Examining mouse left ventricles by immunohistochemistry with anti-CD3e (Fig. 2a), we found that T cells were significantly more abundant in TAC versus sham mice at 4 weeks (Fig. 2b), confirming the mRNA data. T cells react in an antigen-specific manner, involving few specific clones that subsequently expand in number. Thus, we hypothesized that T cells should also be detectable in the heart at an early stage of disease. We thus performed immunohistochemistry analysis on mice at 1 week post-TAC, and indeed we were able to detect T cells (Fig. 2c). We also performed lymphocyte-enriching gradient purification on cardiac suspensions from hearts of mice at 1 week post-TAC, and detected CD3e-expressing cells in the resultant cell populations by flow cytometry (Fig. 2d). Therefore, T cells were present in the hypertrophic myocardium even at an early stage of the pathology.

Studies in the TAC model have identified that cardiac dysfunction can be detected as early as 2 days post-TAC^7^. T cell activation is often initiated at the lymph nodes that drain the site of inflammation. We thus examined via flow cytometry whether, at 2 days post-TAC, T cells were activated in the heart-draining (mediastinal) lymph nodes. We also examined non-draining (inguinal) lymph nodes as well as spleens of the same animals. At day 2, a significant upregulation of the activation marker CD25 could be seen among CD3^+^ T cells in the heart-draining lymph nodes, though not in the more distal, non-draining lymphoid compartments (Fig. 2e; gating strategy shown in Supplementary Fig. 3a). T cell presence in the ailing myocardium could create an opportunity to manipulate their function for therapeutic purposes.

In order to confirm the relevance of our findings for human disease, we examined T cell abundance in cardiac tissue derived from HF patients suffering from primary cardiomyopathy. We examined tissue from patients carrying lamin A/C mutations, which, as previously described^23^, lead to dilated cardiomyopathy and HF. A subset of these carried a second mutation in titin, leading to a more severe dilated cardiomyopathy. We chose these patients as their cardiomyopathy is caused by a non-immunological cause, unlike inflammatory, autoimmune or viral cardiomyopathies^2^. Detection of T cells in the left ventricle of these patients would suggest that presence of T cells is correlated not only with cardiomyopathies initiated by excessive immune responses, but also with cardiomyopathies triggered by non-immune causes. Cardiac samples were obtained during left ventricular assist device (LVAD) placement surgery, attesting to the advanced stage of their cardiac dysfunction^23^. Azan's trichrome collagen staining (Fig. 2f) confirmed presence of fibrosis in these specimens (Fig. 2g). Analysis of T cell abundance via CD3e immunohistochemistry (Fig. 2h) in the same samples revealed the presence of infiltrating T cells (Fig. 2i), similar to hearts of mice at 4 weeks post-TAC. In addition to the above, we also examined samples from patients suffering from aortic stenosis, which leads to HF^24^ and represents the clinical condition that is mechanistically closest to the TAC mouse model. Left ventricles from patients with this form of cardiomyopathy also demonstrated a similarly increased fibrosis (Fig. 2j) and T cell presence (Fig. 2k). Taken together, while only associative, these results further support a link between T cell presence, cardiac fibrosis and pathological hypertrophy.

### T cell costimulation blockade delays HF and reduces its severity

We hypothesized that specific inhibition of T cell function would have a beneficial effect on HF. CTLA4 is one of the inhibitory molecules through which naturally occurring regulatory T cells, as well as pro-inflammatory T cells at the termination of a response, suppress T cell activation under physiological conditions^25^. It blocks the CD80/CD86 costimulation signals that T cells must receive from antigen presenting cells (dendritic cells, B cells or macrophages) in order to become fully activated^14^. CTLA4-Ig fusion protein (abatacept, an FDA-approved drug for rheumatoid arthritis, an autoimmune disease) is a stable, soluble form of CTLA4. We, therefore, tested whether administration of abatacept produced beneficial effects in the TAC model of HF. We treated mice that had been TAC- or sham-operated with three intraperitoneal injections per week of 200 μg of abatacept, for 4 weeks, starting 2 days after the operation. As controls, TAC- and sham-operated mice received PBS, at the same timepoints. Cardiac function was monitored by transthoracic echocardiography (see Supplementary Table 2). Day 2 post-operation was chosen as the first timepoint of treatment as significant cardiac dysfunction (increase in left ventricle thickness) can already be detected at 2 days post-TAC via clinically-relevant diagnostic techniques (echocardiography)^7^.

**Figure 3:**
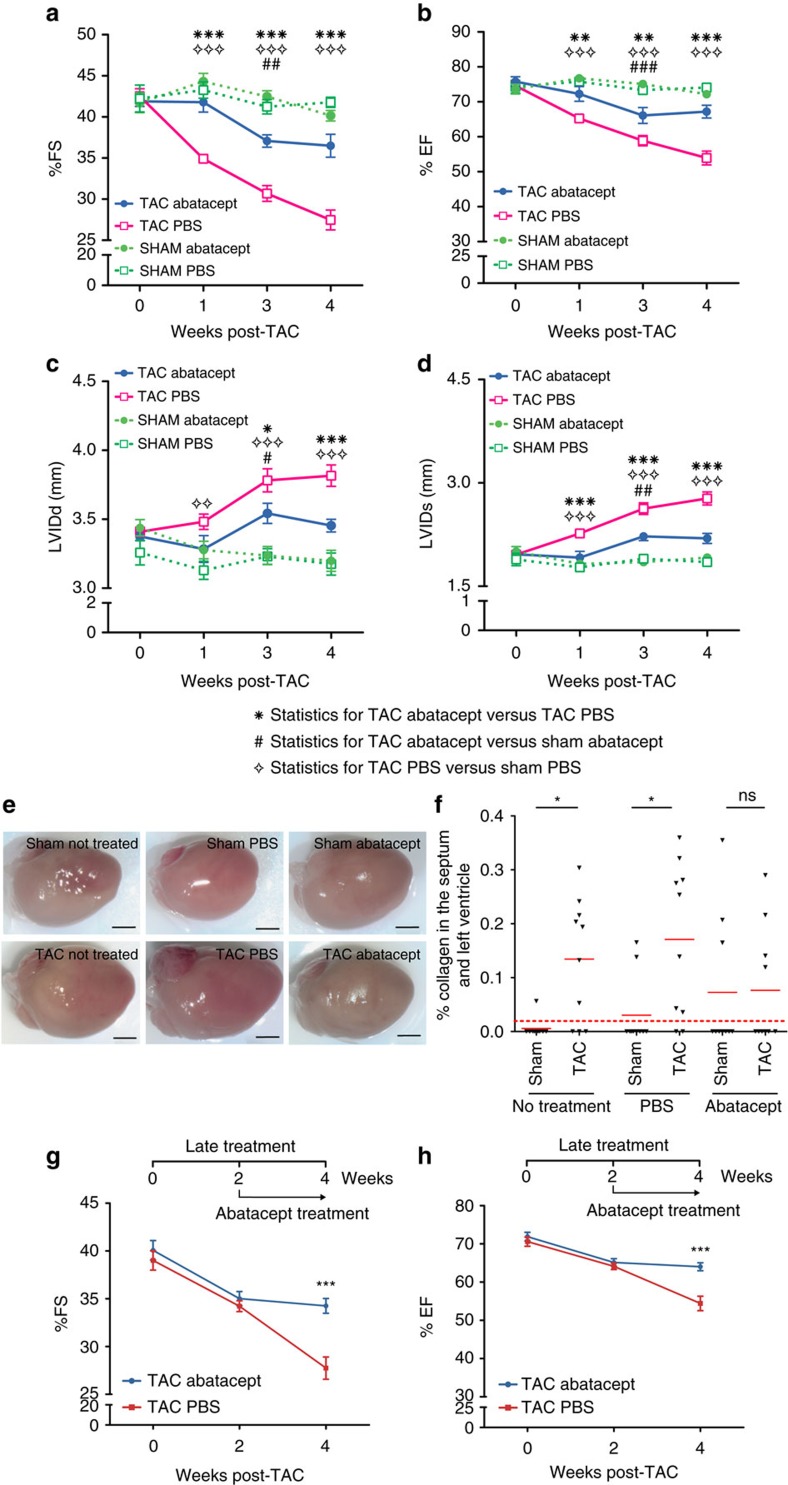
Abatacept blunts progression of cardiac dysfunction in pressure-overloaded mice. Mice underwent TAC or sham operation; 2 days post-operation, the mice were treated with three intraperitoneal injections per week of 200 μg of abatacept or PBS, for 4 weeks. (**a**) Fractional shortening (%FS), (**b**) ejection fraction (%EF), (**c**) left ventricle internal dimension in diastole (LVIDd) and (**d**) left ventricle internal dimension in systole (LVIDs) in TAC- and sham-operated mice at baseline and at time points 1, 3 and 4 weeks after operation, with and without abatacept administration. Data show the mean %FS, %EF, LVIDd and LVIDs for each experimental group at all time-points±s.e.m. (*n*=7–9). Two-way analysis of variance (ANOVA) with Bonferroni post-test: *P* values shown in the panel. Abatacept ameliorates pressure overload-induced cardiac fibrosis in mice. (**e**) Representative macroscopic images of the heart of untreated, PBS-treated and abatacept-injected mice 4 weeks post-sham- or TAC (scale bar=2mm). (**f**) Cardiac sections of untreated, PBS-treated or abatacept-treated, TAC- or sham-operated mice, at 4 weeks post-operation were stained with Azan's trichrome (*n*=2). Five identical regions of interest (ROIs) were applied to all samples. The collagen staining intensity was quantified by image acquisition software; plot points indicate the % of collagen pixels in each ROI. Red bars indicate the mean % collagen in each experimental group. ROIs with a collagen signal higher than zero were considered fibrotic. Fisher's exact tests for the presence or absence of fibrosis were applied to sham versus TAC-operated groups for each treatment category. The dotted red line separates fibrotic from non-fibrotic ROIs. *P value<0.05. (**g**,**h**) Mice underwent TAC, 2 weeks post-operation, the mice were treated with three intraperitoneal injections per week of 200 μg of abatacept or PBS, for 2 weeks. (**g**) Fractional shortening (%FS) and (**h**) ejection fraction (%EF) were measured at baseline and at 2 and 4 weeks after operation. Data show mean of %FS and %EF for each experimental group at all time-points±s.e.m. (*n*=7). Two-way ANOVA with Bonferroni post-test: ***P value<0.001.

PBS-treated TAC-operated mice at 1 and 4 weeks post-operation displayed a significant reduction in cardiac function, expressed as percent fractional shortening (FS) or ejection fraction (EF) compared with sham controls, while abatacept-treated mice had no significant difference in FS or EF from sham controls (Fig. 3a,b). Difference in FS was evident from the first week post-TAC operation, up to the end of the experiment (Fig. 3a); the difference in EF increased in significance with time between the PBS- and abatacept-treated groups (Fig. 3b). Hence by administering abatacept starting from 2 days after TAC surgery, we were able to significantly reduce the extent and delay the progression of degradation of cardiac function. The beneficial effect of abatacept was also evident by analysing other hemodynamic parameters, including end-diastolic and end-systolic left ventricular internal diameter (LVIDd and LVIDs) (Fig. 3c,d). Other measured parameters are reported in (Supplementary Table 2). It should be noted that at 3 weeks post-operation, a transient yet significant difference between abatacept-treated and sham control animals could be seen. At the end of the fourth week, we assessed the morphometric indicators of cardiac hypertrophy: heart weight to body weight ratio (Supplementary Fig. 3b), left ventricle to body weight ratio (Supplementary Fig. 3c), heart weight to tibia length ratio (Supplementary Fig. 3d; representative images in Fig. 3e). Abatacept-treated TAC-operated mice displayed significantly lower hypertrophy than PBS-treated controls, according to most of these parameters. Analysis of myocardial ‘stress genes', hallmarks of cardiac hypertrophy and failure, in the left ventricles by qPCR also showed a significant upregulation of β-Myosin heavy chain (*Mhy7*) (Supplementary Fig. 3e), Brain Natriuretic Peptide (*Nppb*) (Supplementary Fig. 3f) and Atrial Natriuretic Factor (*Nppa*) (Supplementary Fig. 3g) mRNAs for the PBS- but not for the abatacept-treated groups. Thus, abatacept treatment significantly reduces the severity and delays the progression of the cardiac dysfunction caused by the ventricular pressure overload.

We examined sections with Azan's trichrome staining in order to assess the levels of fibrosis^26^. A comparison of collagen intensity in identical regions sampled for all treatment groups identified significant increases in fibrosis levels for all TAC-operated groups except for the mice treated with abatacept (Fig. 3f). These results suggest that the beneficial effect of abatacept is also reflected in protection from cardiac fibrosis, a biological response invariably linked to HF^27^.

Abatacept is based on human CTLA-4 fused with human immunogloblin, but it has been extensively shown to function in mice, due to the high similarity of human and mouse CTLA-4 (refs 28, 29). As human Ig administration could be immunogenic in mice, we included a further set of non-operated mice that received abatacept or an isotype control immunoglobulin (Ig), to assess any effects of the human Ig used in the fusion protein. Neither abatacept alone nor human IgG control injections led to any significant effects in heart function in non-operated animals (Supplementary Fig. 4a,b), suggesting that any alloreactivity to the immunoglobulin had limited effects. Nonetheless, the potential for alloreactivity of the IgG control, in the absence of the immunosuppressive CTLA-4 domain, could possibly worsen the TAC-induced inflammation. For this reason, we chose to use PBS administration rather than IgG administration as a control for our experiments, so as to avoid any deleterious effect on the controls creating the appearance of a stronger therapeutic effect in the abatacept-treated group. Indeed, when we assessed the *in vivo* effect of abatacept in TAC-operated mice, we found that its protective effect appeared to be even more significant when compared with isotype control-treated rather than PBS-treated TAC-operated mice (Supplementary Fig. 4c–f). This confirmed the validity of our choice of controls.

We next wondered whether abatacept treatment would be able to block the progression of cardiac dysfunction if administered only at a late timepoint, when the disease is more advanced. We thus repeated the *in vivo* treatment with abatacept, albeit commencing the first treatment at 2 weeks post-TAC, instead of 2 days post-TAC. As it can be seen (Fig. 3g,h) treatment at a late timepoint was able to significantly block further reduction of FS and EF in treated animals. A significant protective effect was also observed in LVIDs, though not LVIDd (Supplementary Fig. 4g,h). These results demonstrate that even late treatment with the drug may have substantial beneficial effects in limiting the progression of HF.

### Abatacept inhibits T cell and macrophage activation

**Figure 4:**
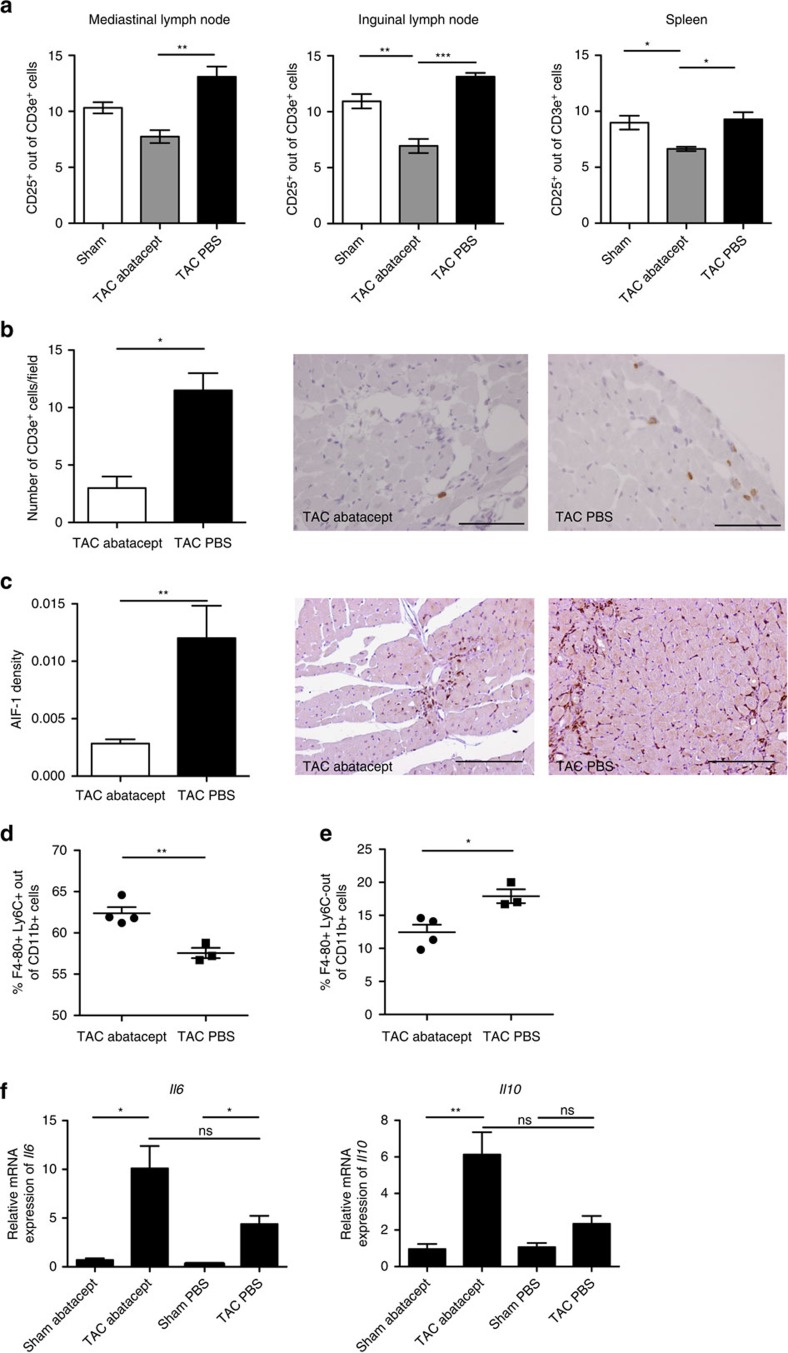
Abatacept administration suppresses the immune response in TAC-operated mice. (**a**) Mediastinal (heart-draining), inguinal lymph nodes and spleens were collected 1 week after TAC or sham-operation, stained and analysed by flow cytometry. Percentage of CD25^+^ out of CD3e^+^ cells are plotted as mean±s.e.m.; sham (white bars), TAC abatacept (grey bars) and TAC PBS (black bars) (*n*=3). One-way analysis of variance (ANOVA) with Tukey's post-test: *P value<0.05; **P value<0.01, ***P value<0.001. (**b**) Statistical analysis of immunohistochemical staining of left ventricles for the T cell marker CD3e in TAC mice at 4 weeks post-operation, treated with abatacept or PBS, and representative images of the staining (brown colouration; original magnification 40 × ; scale bar=50 μm). Number of CD3e^+^ cells is plotted as mean±s.e.m.; TAC abatacept (white bars); TAC PBS (black bars). Unpaired *t*-test; *P value<0.05 (*n*=2). (**c**) Statistical analysis of immunohistochemical staining of left ventricles for the macrophage marker AIF-1 in TAC mice at 1 week post-operation, treated with abatacept or PBS, and representative images of the staining (brown colouration; original magnification 20 × ; scale bar=100 μm). AIF-1 density plotted as mean±s.e.m.; TAC abatacept (white bars); TAC PBS (black bars). Unpaired *t*-test; **P value<0.01 (*n*=2). (**d**,**e**) Cardiac single cell suspensions of TAC operated mice, 1 week after the operation, were stained and analysed by flow cytometry. Percentage of F4-80^+^ Ly6C^+^ out of CD11b^+^ CD45^+^ live cells (**d**) and F4-80^+^ Ly6C^-^ out of CD11b^+^ CD45^+^ live cells (**e**) are plotted as mean±s.e.m.; TAC abatacept (black circles); TAC PBS (black squares). Unpaired *t*-test; *P value<0.05; **P value<0.01 (*n*=4, 3). (**f**) Gene expression analysis (TaqMan real-time qPCR) of the left ventricle of C57BL6/J mice, 1 week after TAC or sham operation, with abatacept or PBS treatment. Bars show relative mean *Il6* and *Il10* expression, internally normalized to 18 s ribosomal RNA expression. Values are mean±s.e.m. (*n*=5, 8). One-way ANOVA, Dunn's post-test: *P value <0.05; n.s., not significant.

Extensive studies have shown that CTLA4-Ig inhibits T cell function by blocking the costimulatory receptors on antigen presenting cells, which are required for the full activation of pro-inflammatory T cells^14^,^30^. The CTLA-4 molecule represents one of the main available mechanisms through which already initiated T cell responses can be physiologically downregulated^31^,^32^. Indeed, we found that *in vitro* abatacept administration to splenocytes inhibited T cell responses (Supplementary Fig. 5a). We, therefore, sought to dissect how abatacept was affecting T cell activation in pathological cardiac hypertrophy. For this, we examined via flow cytometry the expression of activation marker CD25 in T cells at an early timepoint (1 week post-TAC), which is likely to be within the relevant time window for activation events. Abatacept significantly reduced the percentage of CD25^+^ cells among T cells, not only in the heart-draining (mediastinal) lymph nodes, but also in inguinal lymph nodes and spleen (Fig. 4a). This suggests that abatacept exerted a systemic dampening of T cell activation. CD25 expression on the T cells infiltrating the heart could not be reliably assessed due to the low number of T cells found in the heart at 1 week post-TAC, which renders flow cytometric analysis of subpopulations technically challenging. Reduced T cell activation is likely to lead to reduced proliferation and lower T cell numbers at later timepoints. Indeed, at 4 weeks after surgery, the myocardium of abatacept-treated mice displayed significantly fewer infiltrating T cells than PBS-treated mice (Fig. 4b).

Abatacept has also been shown to inhibit T cell-dependent monocyte/macrophage activation and function^33^ and B-cell function^34^, as these cells physiologically provide costimulation to T cells via CD80/CD86. We thus wondered whether abatacept administration in TAC-operated animals led to inhibitory effects on macrophage activation, which has been shown to contribute to cardiac pathology^35^. We assessed via immunohistochemistry the expression of AIF-1 (Iba-1), a marker of T cell-derived macrophage activation^36^,^37^, in the hearts of operated mice, at 1 week post-surgery. In TAC-operated mice, abatacept treatment led to a significant reduction in AIF-1 signal compared to PBS-treated controls (Fig. 4c). Sham-operated mice had negligible signals of AIF-1^+^ cells (Supplementary Fig. 5b). At 4 weeks post-surgery, the difference in AIF-1^+^ macrophages between the TAC-operated groups was minimal (Supplementary Fig. 5c), most likely as the overall levels of AIF-1^+^ macrophages, or indeed total CD11b^+^ innate immune cells (Fig. 1) in TAC-operated mice is reduced at this late stage of the pathology.

We next examined the maturation state of macrophages^38^ in the left ventricles of abatacept or control-treated TAC mice at 1 week post-operation, by flow cytometric analysis. We considered the percentage of Ly6C^+^F4-80^+^ (immature macrophages) or Ly6C^-^F4-80^+^ (mature macrophages) out of CD11b^+^CD45^+^ live single cells (gating strategy shown in Supplementary Fig. 5d). We found that hearts of abatacept-treated animals had significantly higher percentage of immature macrophages (Fig. 4d) and significantly lower percentage of mature macrophages (Fig. 4e), compared with controls.

The above findings suggest that abatacept inhibits T cell activation and infiltration/proliferation, but also targets the activation and maturation state of macrophages in the myocardium.

### The abatacept effect is dependent on IL-10 produced by B cells

The effect of abatacept on T cell activation occurs via the removal of pro-inflammatory, costimulatory signals^32^ on antigen presenting cells^39^. Yet it can additionally be dependent on the production of anti-inflammatory signals, actively inhibiting the pathogenic response^30^,^40^. To investigate this, we examined the presence of immune mediators via real-time qPCR in the left ventricles of treated TAC-operated animals. At 1 week post-operation, a timepoint when abatacept already leads to cardioprotective effects, mRNA expression for the pro-inflammatory cytokine IL-6 was significantly upregulated in both abatacept- and PBS-treated TAC-operated mice (Fig. 4f: *il6*). However, only in abatacept-treated mice could we observe a significant upregulation of mRNA for the cytokine IL-10 (Fig. 4f: *il10*). IL-10 is one of the most potent anti-inflammatory cytokines utilized by the immune system to shut down unwanted or no-longer-needed responses and it has been shown to mediate cardio-protective effects in HF^41^, its effect on cardiomyocyte function being opposite to that of IL-6 (ref. 9). Direct *in vitro* administration of abatacept on cultured neonatal cardiomyocytes did not have any effects on their hypertrophic state (Supplementary Fig. 6a). These findings collectively suggest that abatacept could be mediating anti-inflammatory and subsequent anti-hypertrophic effects via the action of IL-10. As *Il10* was upregulated in abatacept-treated TAC mice, we assessed which subset of immune cells could function as IL-10 sources. We examined the expression of intracellular IL-10 by flow cytometry in splenocytes exposed *in vitro* to abatacept. We found that abatacept induced IL-10 mostly on antigen-presenting cells, the vast majority of which were B cells, while a few IL-10 producing T cells could also be identified (Supplementary Fig. 6b,c).

**Figure 5:**
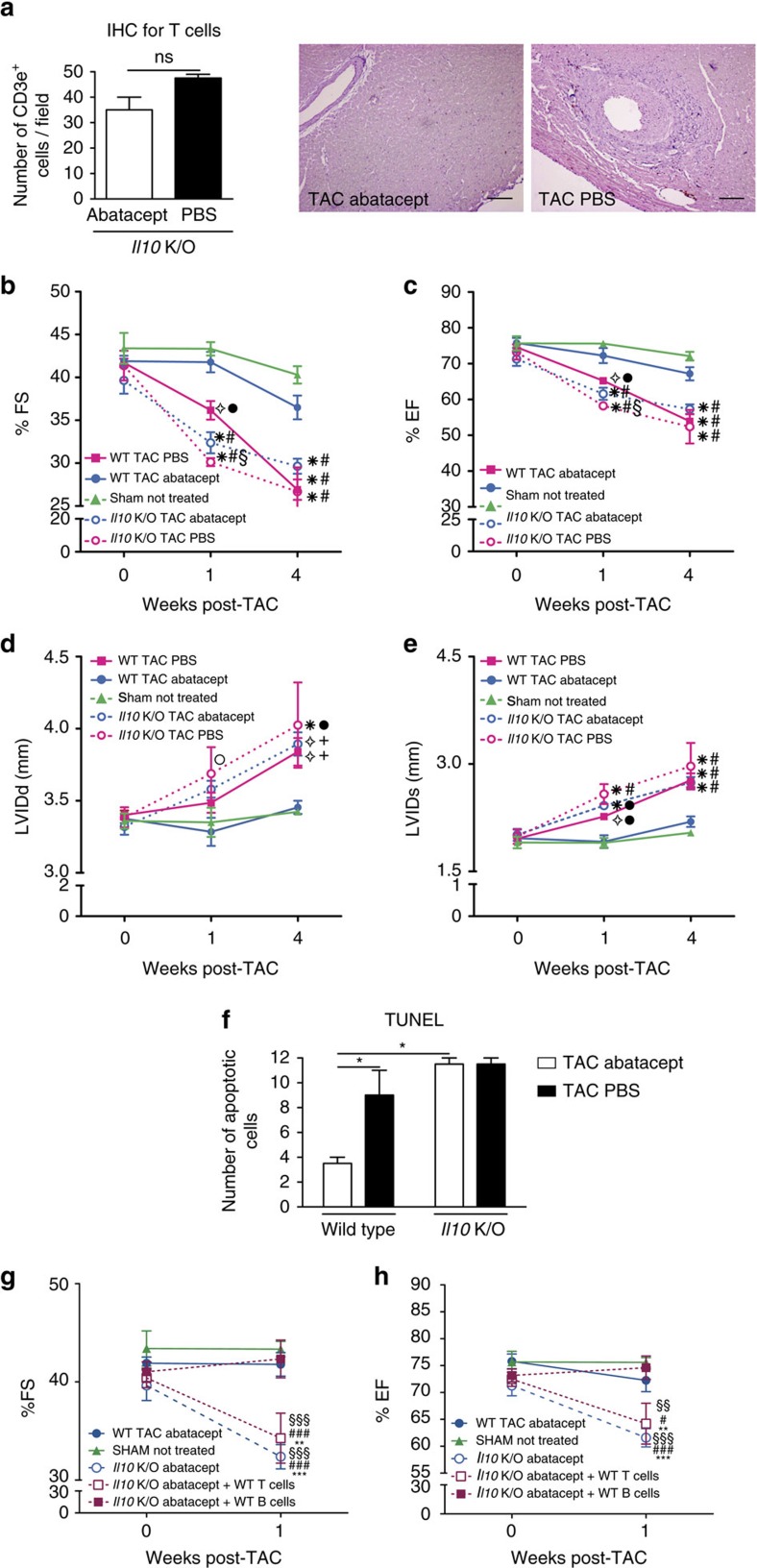
Abatacept attenuates HF through the action of IL-10. (**a**) Immunohistochemical staining of left ventricles for CD3e in TAC-operated *Il10* KO mice treated with abatacept or PBS, 4 weeks post-operation. Mean±s.e.m. (*n*=2). Unpaired *t*-test; ns, not significant. Representative staining for CD3e (brown; original magnification 20 × ; scale bar=100 μm). (**b**–**e**) Heart functionality is not preserved in *Il10* KO TAC-operated mice after abatacept treatment. TAC/sham-operated mice, starting 2 days post-operation, were treated with three intraperitoneal injections per week of abatacept or PBS, for 4 weeks. (**b**) Fractional shortening (%FS). (**c**) Ejection fraction (%EF). (**d**) Left ventricle internal dimension in diastole (LVIDd). (**e**) Left ventricle internal dimension in systole (LVIDs). Mean±s.e.m. (*n*=5–9). Two-way analysis of variance (ANOVA) with Bonferroni post-test; open circle, *P* value<0.05 versus TAC WT abatacept; open four pointed star, *P* value<0.01 versus TAC WT abatacept; *P value<0.001 versus TAC WT abatacept; ^+^*P* value<0.05 versus sham not-treated; closed circle, *P* value<0.01 versus sham not-treated; ^#^*P* value<0.001 versus sham not-treated; ^§^*P* value<0.01 versus TAC WT PBS. (**f**) Abatacept treatment in the presence but not absence of IL-10 reduces cardiomyocyte apoptosis in TAC-operated mice. TUNEL assay staining in slides for cardiomyocyte apoptosis on hearts of treated mice 4 weeks post-TAC, in wild-type and *Il10* KO mice. Mean±s.e.m. of TUNEL-positive cells (*n*=2); white bars, abatacept-treated TAC-operated mice; black bars, PBS-treated TAC-operated mice. Two-way ANOVA with Bonferroni post-test; *P value<0.05. (**g**,**h**) Wild-type B cell but not T cell transfer in *Il10* KO TAC-operated mice restores abatacept therapeutic effects. *Il10* KO mice received wild-type T or B cells. Subsequently, they underwent TAC or sham operation and then treated with abatacept as in **b**–**e**. (**g**) %FS and (**h**) %EF at baseline and 1 week after operation. Mean %FS and %EF for each experimental group at all time-points±s.e.m. (*n*=3–7). Two-way ANOVA with Bonferroni post-test, *statistics for *Il10* KO TAC abatacept; +WT B cells; #statistics for WT TAC abatacept; §statistics for sham not treated.

We thus examined whether IL-10 was necessary for the protective effects of abatacept. We analysed the effect of abatacept on mice deficient for IL-10 (*Il10* KO) subjected to TAC. The hallmark of abatacept function is the suppression of T cell responses^14^,^30^. Interestingly, in *Il10* KO TAC-operated mice, abatacept could no longer inhibit T cell presence in the heart (Fig. 5a), demonstrating that IL-10 is required for the T cell-attenuating, anti-inflammatory effect of the drug. Subsequently, we asked whether IL-10 was necessary for the abatacept-mediated effects on cardiac hypertrophy. Echocardiographic analysis of TAC-operated, *Il10* KO mice confirmed that IL-10 was required for the beneficial effect of abatacept on the heart (Fig. 5b–e). Finally, apoptosis of cardiomyocytes is a hallmark of pathological hypertrophy^26^. While abatacept significantly reduced the extent of cardiomyocyte apoptosis in wild-type TAC-operated mice, this did not occur in *Il10* KO mice, which were refractive to treatment (Fig. 5f).

We thus sought to confirm whether the IL-10 producing cells identified above (that is, mostly B cells, and—to a lesser extent—T cells) could be sufficient to rescue the loss of the protective effect in *Il10* KO animals. To achieve this, we first transferred 2 × 10^6^ wild-type (*Il10*-sufficient) B cells or 2 × 10^6^ wild-type (*Il10*-sufficient) T cells into *Il10* KO recipients. We then performed TAC surgery followed by abatacept or control treatment, starting from day 2 post-operation. Transfer of *Il10* wild-type B cells was sufficient to rescue the loss of the abatacept-mediated protective effect in *Il10* KO TAC-operated mice (Fig. 5g,h: closed squares). On the other hand, transfer of *Il10* wild-type T cells could not rescue the protective effect (Fig. 5g,h: open squares). From this we conclude that IL-10 produced by B cells in response to abatacept must be involved in the mechanism of the abatacept-mediated cardioprotective effect. To assess whether this B cell-mediated effect was dependent on the drug's effect on T cells or whether it could be a direct effect on B cells, we assessed the capacity of splenocytes to produce IL-10 after abatacept administration *in vitro*, in the presence or absence of T cells. We found that the production of IL-10 was unaffected by the absence of T cells (Supplementary Fig. 6d,e), suggesting that the B cell-mediated effect may be direct.

Our results, taken together, suggest that abatacept may protect against the progression of HF by inhibiting the pathogenic immune response mediated by T cells and macrophages, while also directly inducing the beneficial production of anti-inflammatory cytokine IL-10 by B cells.

## Discussion

In this report we demonstrate how abatacept, an FDA-approved drug that inhibits T cell costimulation, reduces severity and delays progression of pressure overload-induced cardiac hypertrophy and fibrosis. Importantly, we were able to demonstrate that the drug could significantly limit the progression of pathology even when administration commenced at a late stage of disease. This was possible because HF pathogenesis is associated with an innate and adaptive immune response. Abatacept blunted this response, and hence inhibited cardiac pathology, via a mechanism dependent on IL-10.

The cardiac inflammation associated with HF is triggered by pro-inflammatory cytokine secretion by stressed cardiomyocytes^3^,^6^,^7^. These cytokines can be used to distinguish between physiological and pathological hypertrophy^42^. We show that immune cell presence can also be used in the same manner. Targeting T cell-mediated responses made it possible to interfere with cardiac remodelling. This is in contrast to unsuccessful attempts to limit pathology by targeting cytokines, which have proven to be more elusive targets^5^,^11^.

A main clinical feature of pathological cardiac hypertrophy is fibrosis. Fibrosis formation in other contexts requires the combined action of Th2 cells and innate immune cells^19^,^43^. In the TAC model we identified an initial M1-polarized innate response, which we speculate subsequently switches to an M2/Th2 polarization. This agrees with studies reporting worse HF in BALB/c compared with C57BL/6 mice, attributed to a Th2-bias of the former strain^13^,^44^. We demonstrated the presence of T cells in cardiac biopsies from human HF patients. Moreover, recent evidence shows that genetic deficiency of T cells improves symptoms in the TAC mouse model^45^,^46^. These findings, collectively, make a strong case for attempting to regulate T cell-mediated responses in order to combat HF.

Immunosuppressive regulatory T cells (Treg) can block deleterious or unwanted responses^25^. Intriguingly, evidence has linked Treg deficiency with chronic HF^47^. We detected the presence of Tregs, via the expression of their genetic marker *Foxp3*, in TAC mice, but only at 8 weeks post-surgery (Supplementary Fig. 6f). This may be an indication of a natural immunosuppressive attempt that occurs too late to block the pathogenic immune response^48^. There have been attempts to utilize Treg adoptive cell therapy in models of HF^49^,^50^. However, cell therapy is a promising procedure that still needs refinement before it can move to clinical use. Treg can also be activated via super-activating anti-CD28 antibodies, which have been utilized in models of cardiac repair after myocardial infarction^51^,^52^. Yet past clinical trials with super-activating anti-CD28 have activated pro-inflammatory memory T cells, with near-lethal consequences for the patients^53^. Searching for a more readily translatable solution, we utilized abatacept, a fusion protein based on CTLA-4. Treg suppress via surface-bound CTLA-4 as well as soluble IL-10 or TGFβ, inhibiting the function of both innate and adaptive immune cells^25^. CTLA-4 inhibits T cell function by blocking the ability of T cells to become costimulated. CTLA4-Ig fusion abatacept is easily administered and already in clinical use to suppress autoimmune responses^14^.

We chose to utilize the TAC mouse model of HF^15^, which leads to Heart Failure with reduced Ejection Fraction. As no model reflecting the characteristics of Heart Failure with preserved Ejection Fraction has been fully consolidated, TAC remains the most commonly used model for the experimental study of HF^54^,^55^. It should be noted that any inflammation induced by TAC surgery *per se* rather than the constriction may not be fully controlled by the sham operation. Having stated this, as Supplementary Fig. 1b suggests, the surgery-induced inflammation in the sham controls is not negligible.

We demonstrated that abatacept reduced the severity of cardiac pathology and delayed the progression of symptoms of overload-derived cardiac pathology. Our aim was to demonstrate that immunity has a contributing (and targetable) role in the development and maintenance of HF. The presence of T cells in biopsies from patients suffering from either lamin A/C cardiomyopathy (associated with Heart Failure with reduced Ejection Fraction, similarly to the TAC model, yet caused by genetic defects), or aortic stenosis (driven by pressure overload, similarly to the TAC model, yet frequently associated with Heart Failure with preserved Ejection Fraction) offers hope for the theoretical applicability of our approach in the clinic. Translation to the human setting will need further exploration.

Abatacept is known to inhibit T cell activation and proliferation^14^ by blocking costimulatory ligands CD80 and CD86 on antigen presenting cells (dendritic cells, B cells and macrophages)^39^,^56^,^57^,^58^. Despite early contrasting data, abatacept has been shown not to act via induction of signals in dendritic cells^59^,^60^. Yet, as it interacts with macrophages and B cells, it is not surprising that it can directly inhibit monocyte/macrophage activation and function^33^,^61^ and B-cell function^34^,^39^,^57^,^58^. The functions of macrophages and B cells affected by abatacept are related to T cell-dependent responses^33^,^34^,^39^, possibly as these functions involve CD80/CD86.

**Figure 6:**
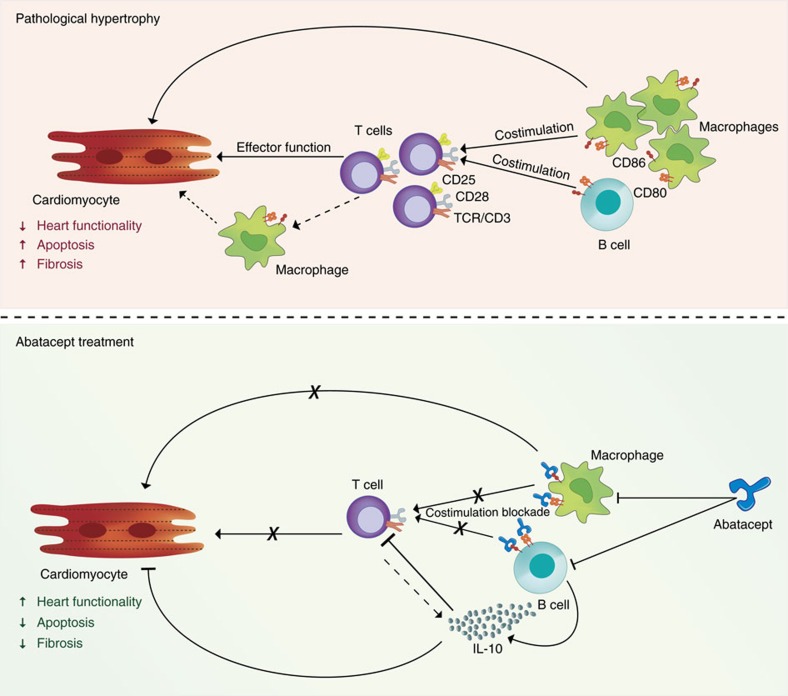
Abatacept blunts cardiac dysfunction by suppressing the immune response. Schematic cartoon of the mechanism of action of abatacept in heart failure. In pathological hypertrophy, T cells are activated (through their TCR) and receive costimulation via CD28 from CD80/CD86-expressing antigen presenting cells (macrophages, B cells, dendritic cells). The full activation of T cells, identified by high levels of CD25, enhances the chronicity of the cardiac inflammatory response. This also involves the proinflammatory action of cardiac macrophages. As a result, there is increased cardiomyocyte apoptosis, fibrosis and reduced heart functionality. During abatacept treatment, the drug blocks CD80/CD86-mediated costimulation by macrophages and B cells, leading to inhibition of T cell activation, proliferation and/or infiltration. The effects on macrophages (which may be both direct and indirect) lead to lower maturation and infiltration. Direct effects on B cells lead to production of anti-inflammatory cytokine IL-10, which may also be produced to a lesser extent by T cells. As a consequence of the effect on T cells, B cells and macrophages, the progression of cardiac pathology is blocked, even if the drug is administered at a late stage. The protective effect is dependent on IL-10 presence.

In agreement to the known mechanisms above, we found that abatacept inhibited T cell responses *in vivo* (Fig. 4a,b), including in heart-draining lymph nodes, where T cell activation appears to be initiated (Fig. 2e). We also observed an inhibition of cardiac macrophage activation and maturation (Fig. 4c–e). Further, we identified the induction of anti-inflammatory cytokine IL-10 (Fig. 4f), which was necessary for the protective effects to occur and which could be produced by B cells after *in vitro* treatment with the drug (Supplementary Fig. 6c). *Il10*-sufficient B cells appeared to be sufficient to rescue the loss of cardioprotective effects in *Il10* KO TAC-operated animals treated with abatacept (Fig. 5g,h). The schematic outline of this combined inhibition of pro-inflammatory T cell/macrophage functions and induction of anti-inflammatory signals in B cells is given in Fig. 6. As shown, T cell^45^,^46^ and monocyte/macrophage^35^ pro-inflammatory function is cardiotoxic. Upon abatacept administration, the mechanisms described above may be acting in parallel. Several caveats must be mentioned: the finding that immune-mediated events can drastically change the outcome of disease does not render inflammation the only aspect that can regulate HF pathology. Second, it should be noted that IL-6 is produced by stressed cardiomyocytes, initiating the inflammatory response that accompanies HF^3^,^6^,^7^,^9^. Our data suggest that inhibition of T cell and macrophage function, which lie downstream of the initial inflammation, triggers compensating anti-inflammatory IL-10 expression but may not be significantly affecting the IL-6 production by cardiomyocytes. Finally, abatacept has been shown to induce regulatory T cells^62^, yet we did not observe any significant induction of *Foxp3* mRNA expression in our system (Supplementary Fig. 6g).

The benefit conferred by abatacept treatment may be that it targets T cell costimulation and thus their optimal activation. T cell activation could be relevant for the chronicity^12^ of the underlying cardiac disease. As a drug already in clinical use, abatacept may be more translationally relevant than other means of targeting T cells currently being explored for the treatment of HF. Further, targeting costimulation requires the targeting of CD80/CD86-bearing macrophages and B cells, which contributes to the therapeutic effect, affecting T cell-associated B cell and macrophage responses.

IL-10 is directly cardioprotective and antifibrotic^19^,^41^. IL-10 was necessary for the cardioprotective effects of abatacept, and for the suppression of T cell expansion (Fig. 5a). Yet IL-10 acts downstream of the administration of abatacept. Thus, the regulation of IL-10 induction will be dependent on localization and abundance of the targets of the drug. Abatacept, even when B cells and macrophages are its direct targets, is known to affect only T cell-associated responses^33^,^34^,^39^. Abatacept did affect T cell activation systemically (as shown in Fig. 4a) but, extrapolating from the data in autoimmune pathologies cited above, it may not affect T cell-independent innate immune responses, even if its action is dependent on IL-10. IL-10 is a very potent anti-inflammatory cytokine; clinical trials for its use have yet to succeed^63^. Its direct administration could possibly block T-independent responses resulting in more severe immunosuppression. Thus, we speculate that abatacept, given its proven clinical safety profile, may be more translationally relevant compared with IL-10 administration, as a potential HF therapy tool.

Taken together, our findings demonstrate how an FDA-approved drug inhibiting pro-inflammatory T cell function, along with effects on macrophages and B cells, yields significant therapeutic benefits in a model of HF. This occurs as an adaptive immune response may be causatively linked to the pathogenesis of pressure overload-induced HF. An immune response driven by cardiac pressure overload could be an unwanted consequence of an immune system evolved to deal with pathogen infections. It may be that the body cannot distinguish between infection- and pressure overload-induced stress signals, and hence initiates a deleterious response. Fortuitously, the link between immunity and HF also creates an opportunity: validated therapies for treating immune-mediated ailments exist and are already in clinical use. They could be repurposed as potential tools in the fight against HF, paralleling the rationale of recent promising studies in other pathologies^64^.

## Methods

### Animals

All procedures were performed in compliance with national and EU legislation, and Humanitas Clinical and Research Center and Norwegian University of Science and Technology regulations.

### Transverse aortic constriction (TAC)

Procedures were performed according to ref. 15. In detail, TAC was performed on 8–10-week-old male C57BL/6 J mice (Charles River, France) and on 8–10-week-old male C57BL6/J *Il10* KO mice (Jackson Laboratories, US). All animals were screened before operation via echocardiography to establish their baseline. Mice were anaesthetized by intraperitoneal injection of a mixture ketamine (100 mg kg^−1^) and xilazine (10 mg kg^−1^). The chest cavity was opened by a small incision at the level of the first intercostal space. After isolation of the aortic arch, a 8–0 Prolene suture was placed around the aorta and a 27G needle was laced in between. The needle was immediately removed to produce an aorta with a stenotic lumen. The chest cavity was then closed with one 6–0 nylon suture and all layers of muscle and skin closed with 6–0 continuous absorbable and nylon sutures, respectively. A sham group, undergoing surgery without aortic banding, was used as control.

### Echocardiography

A Vevo 2100 high-resolution *in vivo* imaging system (VisualSonics Fujifilm) with a MS550S probe ‘high frame' scanhead was used for echocardiographic analysis. Mice were anesthetized with 1.0% isoflurane for M-mode imaging. Pressure gradients (60 to 90 mm Hg), an index of biomechanical stress, were determined by echo Doppler on all animals that underwent TAC surgery.

### Abatacept treatment

Starting 2 days or 2 weeks after TAC/sham surgery, mice were intraperitonally injected with either 100 μl PBS, 200 μg Human IgG Isotype Control (Novus) or 200 μg CTLA-4 Ig (Abatacept) in 100 μl of PBS, three times a week, for up to 4 weeks. Abatacept is a human CTLA-4-Ig fusion, though due to the high (75%) similarity between human and mouse CTLA-4, it also functions in mouse^28^,^29^,^65^,^66^,^67^. These studies demonstrated *in vivo* efficacy (in different pathological contexts) using a dose range 100-400 μg per mouse, in most cases administered every 2 days. These studies, collectively, identify a range of abatacept dosing that is functional in mouse. As 200 μg per mouse every 2 days was both the median dose of the published mouse studies, as well as (at about 8 mg kg^−1^) very similar to the human dose used in Rheumatoid Arthritis patients (8–10 mg kg^−1^), we selected this dose as the most ‘translationally relevant'.

### Adoptive transfer of wild-type T and B cells in IL10 KO mice

Wild-type B and T cells were isolated from 10–12-week-old male C57BL6/J male mice, respectively, with B Cell Isolation Kit and Pan T Cell Isolation Kit II (Miltenyi Biotec) on an AutoMACS. Purity was assessed by staining with anti-mouse CD3ɛ (145-2C11, BioLegend) or anti-mouse CD19 (eBio1D3, eBioscience), and analysed by flow cytometry. C57BL6/J *Il10* KO male mice, before basal echocardiography screening, were injected intravenously with 2 × 10^6^ WT T or B cells. Mice underwent TAC surgery and were injected with abatacept starting on day 2 after surgery.

### Human biopsies

The severe cardiomyopathy patient samples (HF LVAD) were obtained from patients suffering from lamin A/C mutations, causing dilated cardiomyopathy and HF (HF LVAD 1M). A subset of these carried a second mutation in titin (HF LVAD 2M), leading to a more severe dilated cardiomyopathy. All samples were obtained after informed consent according to the study protocols approved by the ethics committee of the University Hospital of Verona^23^. Aortic stenosis ventricular samples were also obtained after informed consent according to the study protocols approved by Humanitas Research Hospital ethics committee.

### Quantitative reverse transcription PCR analysis

Left ventricles were snap frozen in liquid nitrogen after collection and stored at −80 °C. Tissues were homogenized in 1 ml of PureZol RNA isolation reagent (Biorad) with GentleMACS and GentleMACS M Tubes (Miltenyi Biotec). After isolation of the aqueous phase with chloroform, RNA was extracted using RNeasy Mini Kit (Qiagen). The same amount of RNA was retrotranscribed with the High Capacity cDNA Reverse Transcription kit (Applied Biosystems). Real-time qPCR reactions were performed using TaqMan Probes and TaqMan Universal Master Mix on a REALTIME AB 7900HT cycler (all Applied Biosystems). The following TaqMan gene expression assays were used: Rn18S (Mm03928990_g1) as internal control, *Cd3e* (Mm005996484_g1), *Foxp3* (Mm00475162_g1), *Itgam* (Mm00434455_m1), *Tnfα* (Mm00443260_g1), *Il4* (Mm00445259_m1), *Il17* (Mm00439618_m1), *Ifng* (Mm01168134_m1), *Tgfb1* (Mm01227699_m1), *Il10* (Mm00439614_m1), *Il6* (Mm00446190_m1), *Il1b* (Mm00434228_m1), *Ccl2* (Mm00441242_m1), *Ccl4* (Mm00443111_m1), *Ccl5* (Mm01302427_m1), *Cxcl10* (Mm00445235_m1), *Cxcl11* (Mm00444662_m1). Expression of genes encoding for Brain Natriuretic Peptide (*Nppb*), Atrial Natriuretic Factor (*Nppa*) and Myosin heavy chain β (*Myh7*) expression was tested with primers (IDT) using Sybr Select Master Mix (Applied Biosystems) on a ViiA7 (Applied Biosystems) instrument. The sequences are listed in Supplementary Table 3.

### Transgenic Akt (Akt Tg) mice

Male Akt Tg mice^22^, which constitutively overexpress the active E40K Akt mutant (Akt-E40K) were used at 8 weeks of age.

### Exercise-trained mice

BKS.Cg-m +/+ Lepdb/+db mice^67^ are heterozygous for the leptin receptor mutation but display a wild-type metabolic phenotype when fed on a normal diet. We utilized 8-week-old male mice that were arbitrarily assigned to one of two groups: sedentary and exercise trained 70 min per day, 5 days per week, for 8 weeks. The training was performed as running on an inclined (25°) treadmill, starting with 10 min warm-up at ∼50% running speed before 60 min interval training alternating between 4 min at 85–90% of maximal oxygen uptake and 2 min at ∼50% running speed. Training speed was adjusted at least weekly in order to keep the same relative training intensity. Before and after the intervention period, the mice performed an individualized ramp (90–120 s on each step) treadmill protocol on an inclined (25°) treadmill in a metabolic chamber to determine maximal oxygen uptake. Due to the difference in genetic background (BKS), all analyses of these mice were performed comparing them to their matching controls, so as to avoid genetic background-specific effects.

### Immunohistochemical analysis

Mouse heart samples were fixed in 4% formalin at 4 °C, paraffin-embedded and sectioned at 4 μm. The slides were stained with Azan's trichrome for collagen (BioOptica). Slide images were digitalized and five fields for mouse sections and ten fields for human biopsies analysed to quantify fibrosis, with an image analysis program (ImageJ). Cardiac fibrosis was assessed by measuring the Azan's trichrome-stained area as a percentage of total myocardial area. For immunohistochemistry analysis sample sections on slides were deparaffinized and hydrated through a descending scale of alcohols. Antigen retrieval was performed using DIVA (Biocare Medical) for mouse samples and W-Cap (Biocare Medical) or EDTA 0,5M pH8 (Sigma Aldrich) for human samples. Sections were cooled and then washed with PBS (Lonza) containing 0.05% Tween 20 (Sigma). Endogenous peroxidase was blocked by incubation with Peroxidase I (Biocare Medical) for 20 min at room temperature (RT) and nonspecific sites were blocked with Rodent Block and Background Sniper (Biocare Medical) for mouse and human samples respectively 20 min at RT. The sections were then incubated for 1 h at RT with rat anti-human CD3 (Serotec) diluted 1:1,000 or AIF-1 (Wako) diluted 1:250 or polyclonal rabbit anti-human CD3 (Dako) diluted 1:50, washed, and incubated for 30 min at RT with rat-on-mouse HRP polymer (Biocare Medical) or with Mach1 HRP polymer (Biocare Medical) or with Envision+System anti-rabbit HRP (Dako). Finally, sections were incubated with DAB (Biocare Medical), counterstained with haematoxylin, dehydrated through an ascending scale of alcohols and xylene, and mounted with coverslips using Eukitt (Fluka). All samples were observed and photographed with a microscope Olympus BX53 with a digital camera.

### TUNEL assay on mouse heart samples

Sample sections on slides were deparaffinized and hydrated through a descending scale of alcohols and TUNEL assay was performed (Click-it plus TUNEL assay C10617, Life technology).

### *In vitro* stimulation of splenocytes with abatacept

Total splenocytes were purified from spleens of 8-week-old C57BL/6 J mice. T cells were depleted using magnetic beads on an AutoMACS (Miltenyi Biotec). Total splenocytes or T cell-depleted splenocytes were stimulated with 2 μg ml^−1^ of anti-CD3 and/or 5 μg ml^−1^ LPS (Sigma Aldrich) and cultured with 20 μg ml^−1^ abatacept, IgG isotype control or nothing. After 48 or 72 h of culture, Brefeldin A (eBioscience) was added during the last 4 h of culture and splenocytes were prepared for fluorescence-activated cell sorting analysis.

### Neonatal cardiomyocytes treated with abatacept *in vitro*

Hearts were collected from 1–2-day-old CD1 pups and digested with collagenase. Cardiomyocytes were then separated from fibroblasts by preplating twice for 1 h and through centrifugation. Cardiomyocytes were than plated over gelatin, serum-starved and treated with 100 μM phenylephrine. Four hours after the addition of phenylephrine, 20 μg ml^−1^ of abatacept were added to the culture for 44 h. Cardiomyocytes were harvested in PureZOL (Biorad) for RNA extraction and gene expression analysis.

### Flow cytometry

Single cell suspensions from spleens and lymph nodes were obtained via passing through 70 μm cell strainers in cold PBS^−/−^. Hearts were collected and digested with Liberase TM (Roche). Erythrocytes were removed with lysis buffer (BD Biosciences) from spleen and heart cell suspensions. Cells were stained at the following dilutions of stock reagents: Live/dead Aqua Fluorescent Reactive Dye 1:1,000 (Life Techonologies), anti-mouse CD16/32 1:100 (2.4G2, BD Pharmigen), anti-mouse 1:100 CD45 (30-F11, eBioscience), anti-mouse 1:100 CD3ɛ (145-2C11, BioLegend), anti-mouse 1:100 CD19 (eBio1D3, eBioscience), anti-mouse 1:100 CD11b (M1/70, Biolegend), 1:100 CD11c (Bu15, eBioscience), F4/80 1:100 (CI:A3-1, Serotec) in Supplementary Fig. 5d, F4/80 1:100 (BM8, eBioscience) in Fig. 5d,e and Supplementary Fig. 5c, IL-10 1:80 (JES5-16E3, eBioscience), FoxP3 1:100 (FJK-165, eBioscience), Ly6C 1:100 (HK1.4, eBioscience) or anti-CD25 1:100 (PC61.5, eBioscience). An eBioscience intracellular staining kit was used were applicable. Samples were acquired on a fluorescence-activated cell sorting Canto II (BD) and analysed with FlowJo10.

### Statistics

Statistical analysis was performed in GraphPad Prism. All data sets were tested for normal distribution with normality tests before proceeding with parametric or non-parametric analysis. Grubb's test was performed in order to exclude spurious outliers. Statistical significance was tested using unpaired *t*-test, one-way analysis of variance (ANOVA) with Tukey post-test and two-way ANOVA with Bonferroni post-test for data sets with normal distributions. Statistical significance was tested with Mann–Whitney test and one-way ANOVA with Dunn's post-test for data sets without a normal distribution. Fisher's exact tests were used in the analysis of collagen deposition, testing for the presence or absence of collagen stain.

### Data availability

All the relevant data are available within the manuscript and from the authors upon request.

## Additional information

**How to cite this article:** Kallikourdis, M. *et al*. T cell costimulation blockade blunts pressure overload-induced heart failure. *Nat. Commun.* **8,** 14680 doi: 10.1038/ncomms14680 (2017).

**Publisher's note:** Springer Nature remains neutral with regard to jurisdictional claims in published maps and institutional affiliations.

## Supplementary Material

Supplementary Information Supplementary Figures and Supplementary Tables.
