# Supplementary material for: T cell costimulation blockade blunts pressure overload-induced heart failure
Source: Nat Commun. 2017 Mar 6;8:14680. doi: 10.1038/ncomms14680 (PMC5343521; doi:10.1038/ncomms14680)
Supplement: Supplementary Information — Supplementary Figures and Supplementary Tables. [file ncomms14680-s1.pdf]

a

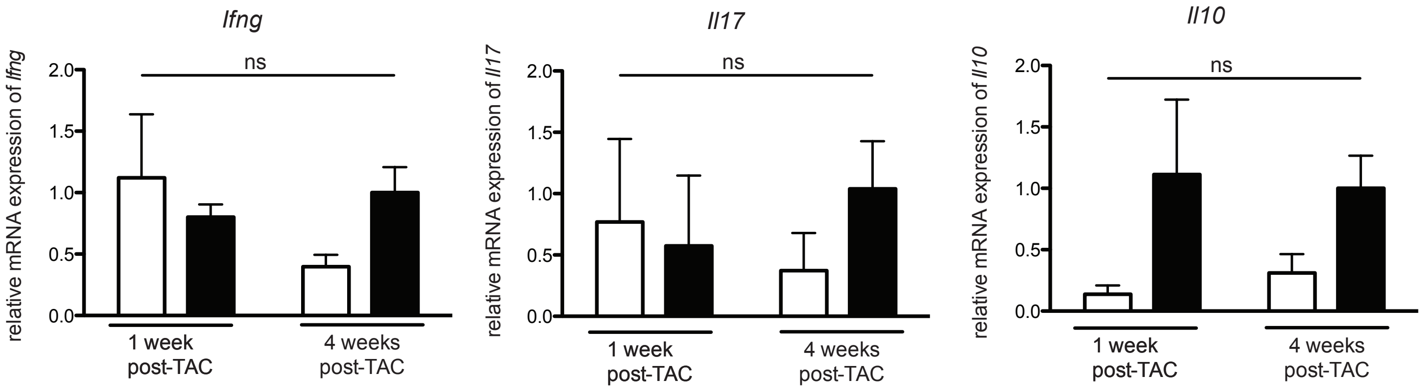

b

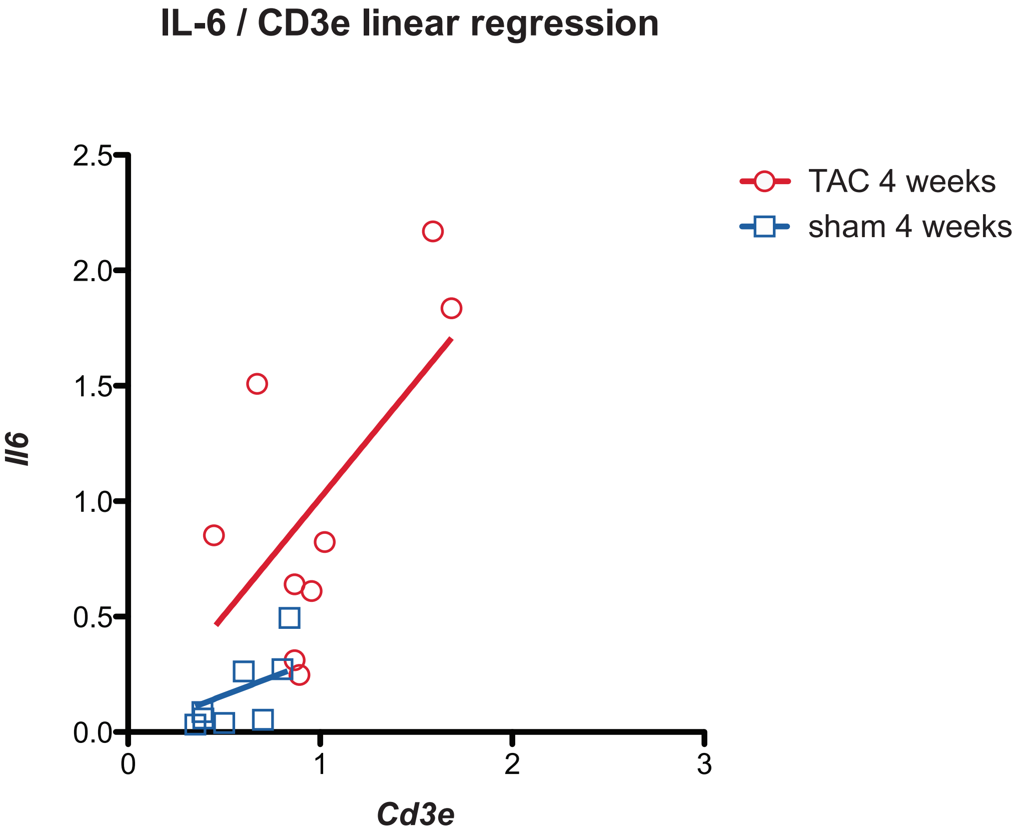

Supplementary Figure 1

(a) Characterization of the inflammatory signature in hypertrophic left ventricle of mice. Gene expression analysis (TaqMan real-time qPCR) of mediators of inflammation within the left ventricle of C57BL6/J mice. Relative mRNA expression in sham-operated control mice (white bars) and TAC-operated mice (black bars) at 1 and 4 weeks after surgery, internally normalized to 18s rRNA expression. *Ifng, Il17* and *Il10.* Values are mean ± SEM (n=7-9). Two-way ANOVA, Bonferroni post-test; ns, not significant. **(b) Linear regression of IL-6 and CD3e mRNA expression in left ventricles of TAC and sham-operated mice. red circles and line:** Linear regression between *Cd3e* and *Il6* expression in left ventricle of C57BL6/J TAC-operated mice 4 weeks after operation (n=9). Linear regression test; p value = 0.0002. **blue squares and line:** Linear regression between *Cd3e* and *Il6* expression in left ventricle of C57BL6/J sham-operated mice 4 weeks after operation (n=7). Linear regression test; p value = 0.0037.

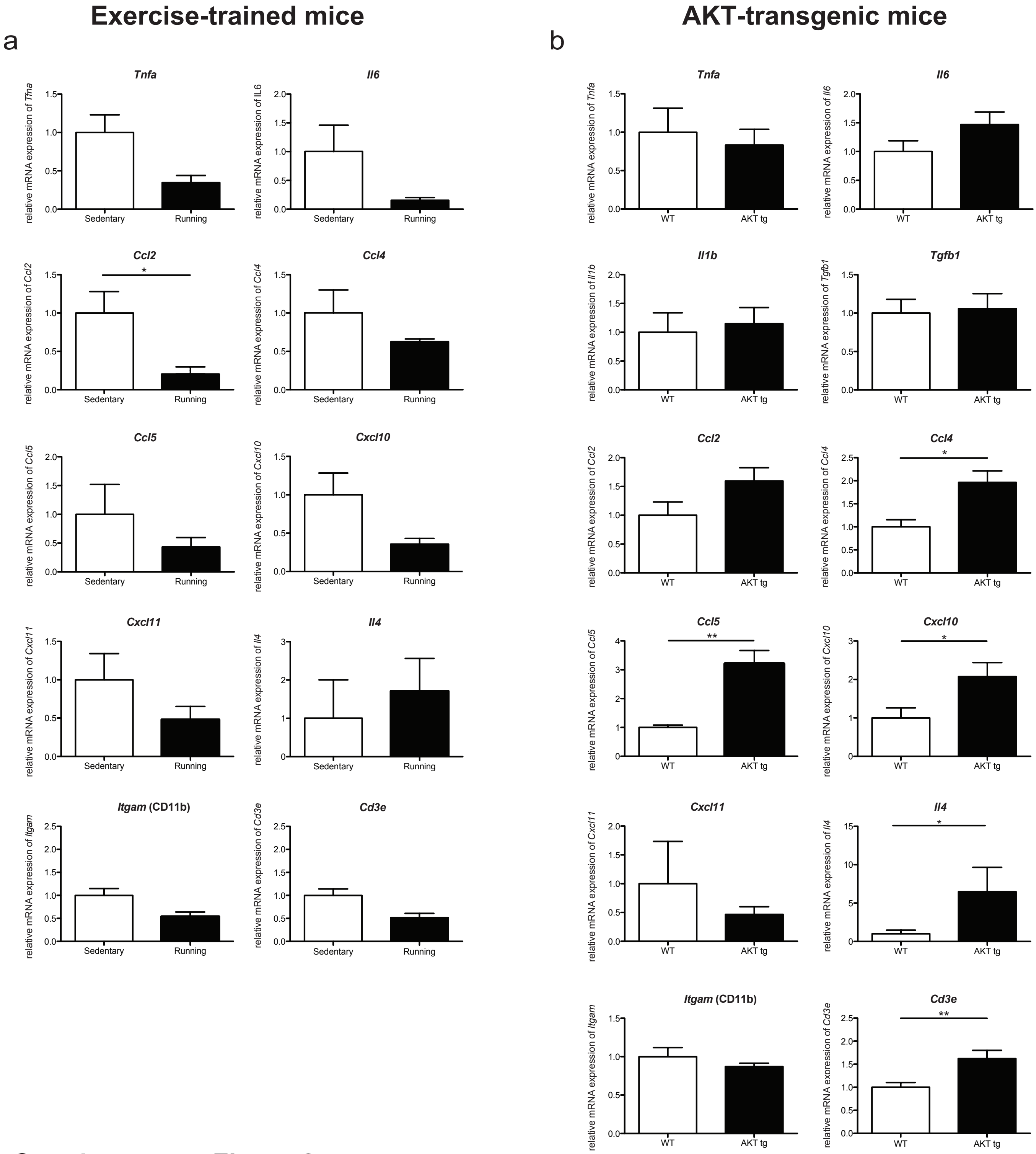

Supplementary Figure 2

Immune mediators in models of physiological hypertrophy (a) Gene expression analysis of mediators of inflammation in the left ventricle of exercise-trained mice (running) compared to sedentary mice (sedentary). Bars show relative mean mRNA expression, internally normalized to 18s rRNA expression. The expression of *Tnfa, Il6, Ccl4, Ccl5, Cxcl10, Cxcl11, Il4, Itgam* (CD11b), or *Cd3e* did not change significantly, whilst *Ccl2* significantly decreased in the trained mice group. Values are mean ± SEM (n=3). Unpaired t-test: *, p-value <0.05. **(b)** Gene expression analysis of inflammatory mediators by TaqMan real-time qPCR in the left ventricle of 8-week-old Akt transgenic mice (Akt-Tg, white) (n=6) compared to wild-type mice (WT, black) (n=7). Bars show relative mean mRNA expression internally normalized to 18s rRNA expression. The relative expression of *Tnfa, Il6, Il1b, Tgfb1, Ccl2, Cxcl11*, and the innate cell marker *Itgam* (CD11b) did not increase, whilst *Ccl4, Ccl5, Cxcl10, Il4,* and *Cd3e* increased significantly. Values are indicated as mean ± SEM. Mann-Whitney test: *, p-value <0.05; **, p-value <0.01.

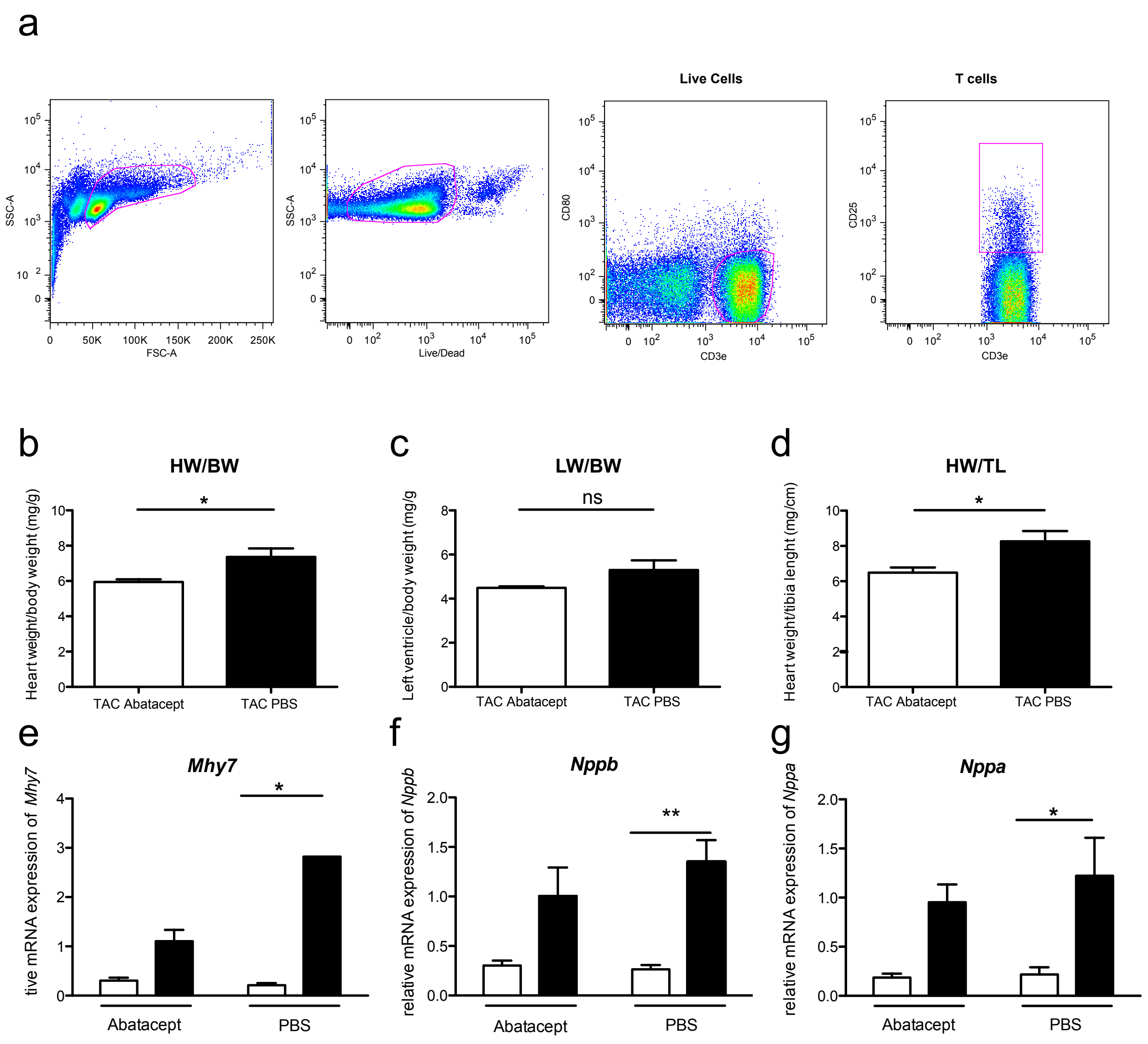

Supplementary Figure 3

Gating strategy for FACS analysis of T cell activation. (a) Cells were gated on forward and side scatter, and dead cells were excluded. The $CD80^{+}$ population corresponded to autofluorescent cells, and hence was excluded. CD3e-expressing cells were selected and CD25 expression was assessed. **Progression of cardiac hypertrophy is limited by abatacept. (b)** Heart weight body weight (mg/g), **(c)** left ventricle body weight (mg/g), **(d)** heart weight tibia length (mg/cm) ratios in TAC-operated mice treated with abatacept or PBS, 4 weeks post operation. Values are indicated as mean ± SEM (n=5-7). Unpaired t-test: *, p-value <0.05. Relative gene expression by real-time qPCR internally normalized to 18S for genes expressing **(e)** β-myosin (*Mhy7*), **(f)** brain natriuretic peptide (*Nppb*) and **(g)** atrial natriuretic factor (*Nppa*) in the same mice. Values are mean ± SEM (n=4-7). Two-way ANOVA with Bonferroni post-test; *, p-value <0.05; **, p-value <0.01.

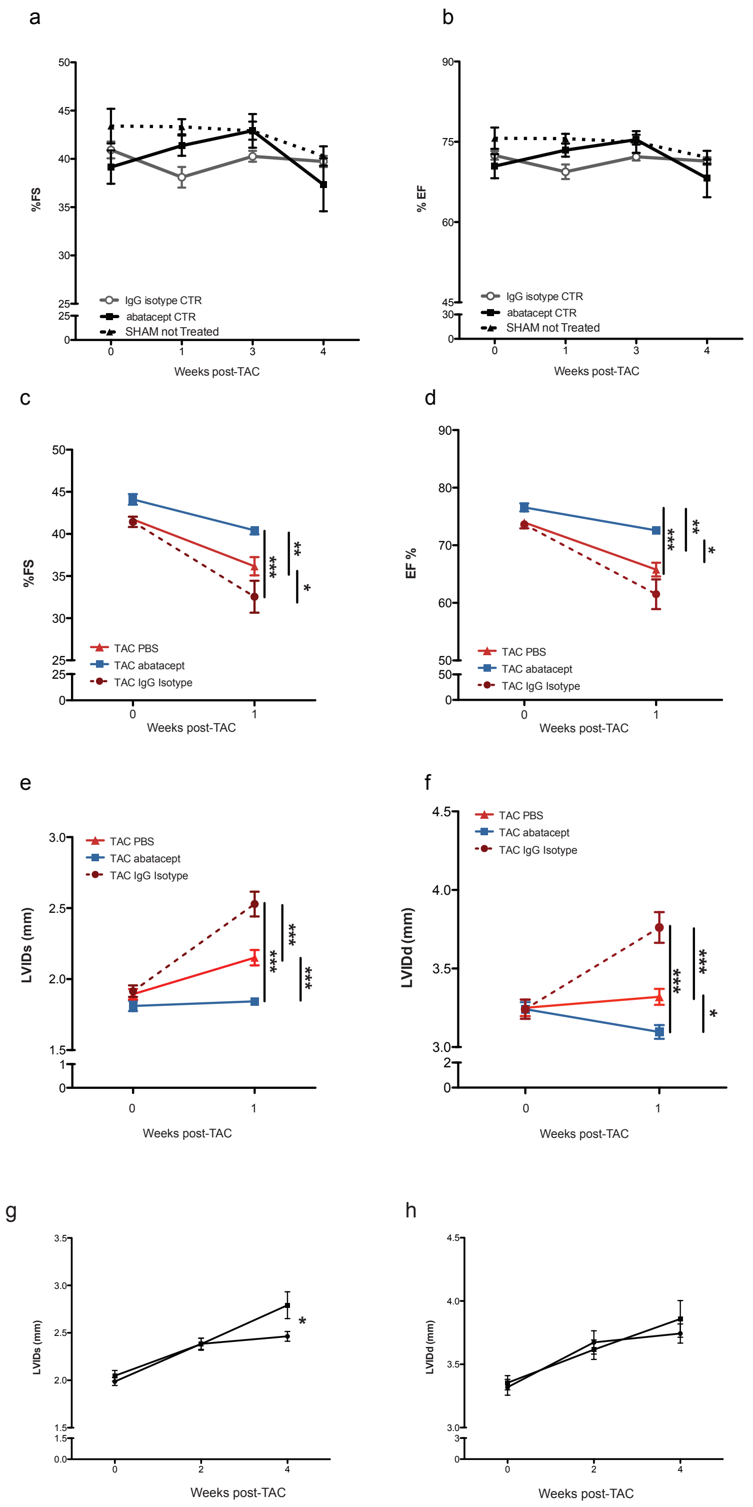

Supplementary Figure 4

Heart functionality is unchanged in non-operated mice after abatacept or human IgG isotype treatment. (a) Fractional shortening (%FS) and **(b)** ejection fraction (%EF) in non-operated mice treated with abatacept or human IgG isotype control, or sham-operated, non-treated mice (included for comparison). Values are mean ± SEM (n=3–4). Two-way ANOVA with Bonferroni post-test; no significant differences observed. **(c)** Fractional shortening (%FS), **(d)** ejection fraction (%EF), **(e)** left ventricle internal dimension in systole (LVIDs) and **(f)** left ventricle internal dimension in diastole (LVIDd) in TAC-operated mice treated with abatacept, PBS or human IgG isotype control for 1 week starting 2 days after operation. Values are mean ± SEM (n=6-9). Two-way ANOVA with Bonferroni post-test; *, p value <0.05; **, p value <0.01; ***, p value <0.001. **(g)** left ventricle internal dimension in systole (LVIDs) and **(h)** left ventricle internal dimension in diastole (LVIDd) in TAC-operated mice treated with abatacept or PBS for 2 weeks starting 2 weeks after operation. Values are mean ± SEM (n=7-9). Two-way ANOVA with Bonferroni post-test; *, p value <0.05.

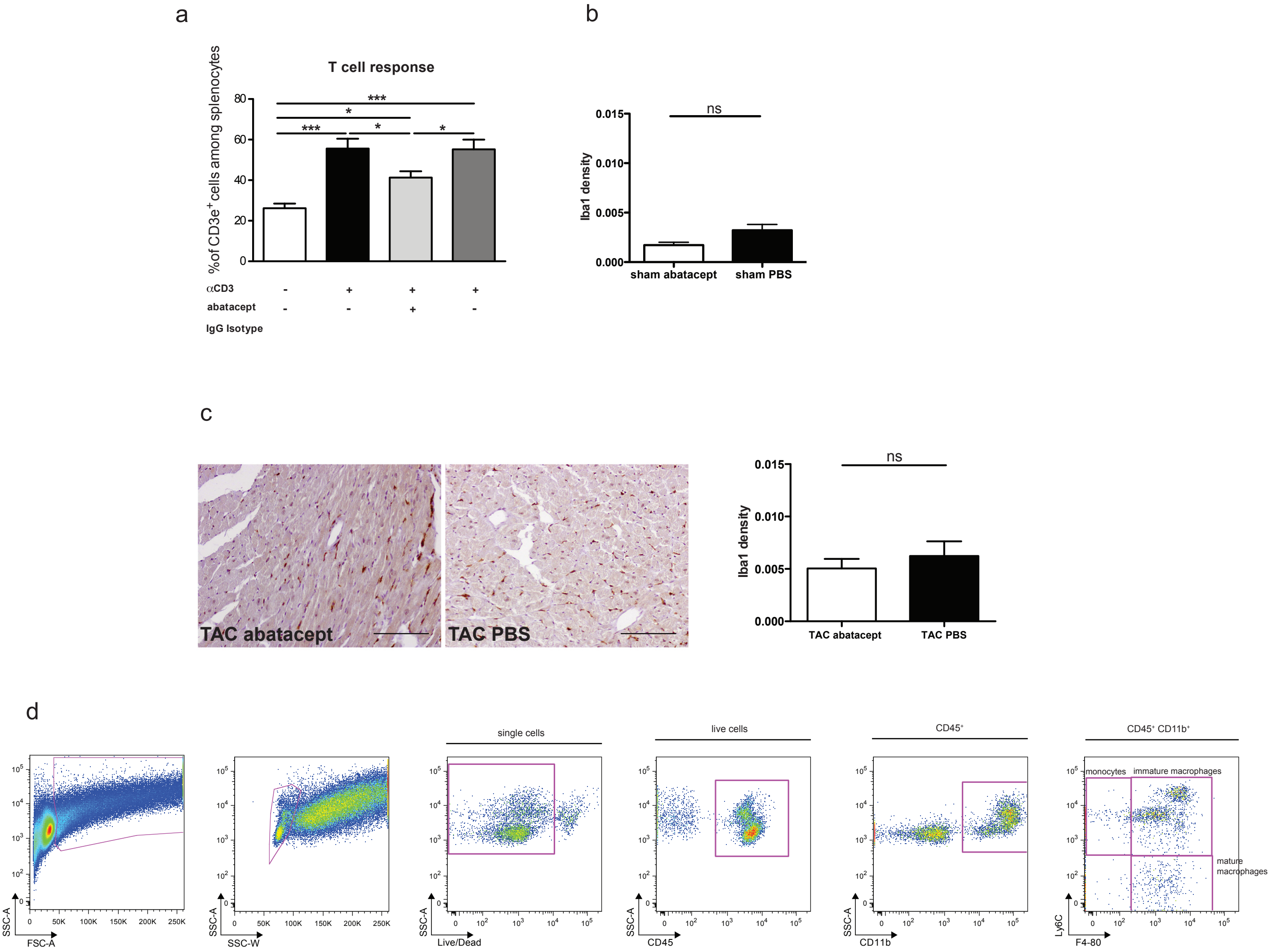

Supplementary Figure 5

Abatacept attenuates T cell responses *in vitro* (a) Total splenocytes of 8 week-old C57BL/6J mice activated with anti-CD3 and cultured with 20µg/ml abatacept or IgG isotype control for 72 hours were analyzed by FACS for CD3e expression. Bars show mean ± SEM of 4 independent experiments (n=4). One way-ANOVA repeated measures test with Tukey's post-test; *, p-value <0.05; ***, p-value <0.001. **(b)** Statistical analysis of AIF-1(Iba1) immunohistochemical staining in left ventricles of sham-operated mice injected with abatacept (white bar) or PBS (black bar) at 4 weeks post-operation. AIF-1 density plotted as mean ± SEM. Unpaired t-test. (n=2). **(c)** Representative images and statistical analysis of AIF-1 immunohistochemical staining (brown coloration; original magnification 20X; scale bar = 100 µm) in left ventricles of TAC mice at 4 weeks post-operation, treated with abatacept or PBS. AIF-1 density plotted as mean ± SEM; TAC abatacept (white bars); TAC PBS (black bars). Unpaired t-test. (n=2). **(d)** Gating strategy for flow cytometry analysis of cardiac single cell suspensions from TAC operated mice 1 week after operation, corresponding to data in **Fig. 4d,e**. Cells were gated based on forward and side scatter, doublets were excluded and live single cells were gated. CD45 and CD11b-expressing cells were selected and identified on the basis of Ly6C and F4-80 expression: Ly6C$^+$ F4-80$^-$ monocytes, Ly6C$^+$ F4-80$^+$ immature macrophages and Ly6C- F4-80$^+$ mature macrophages.

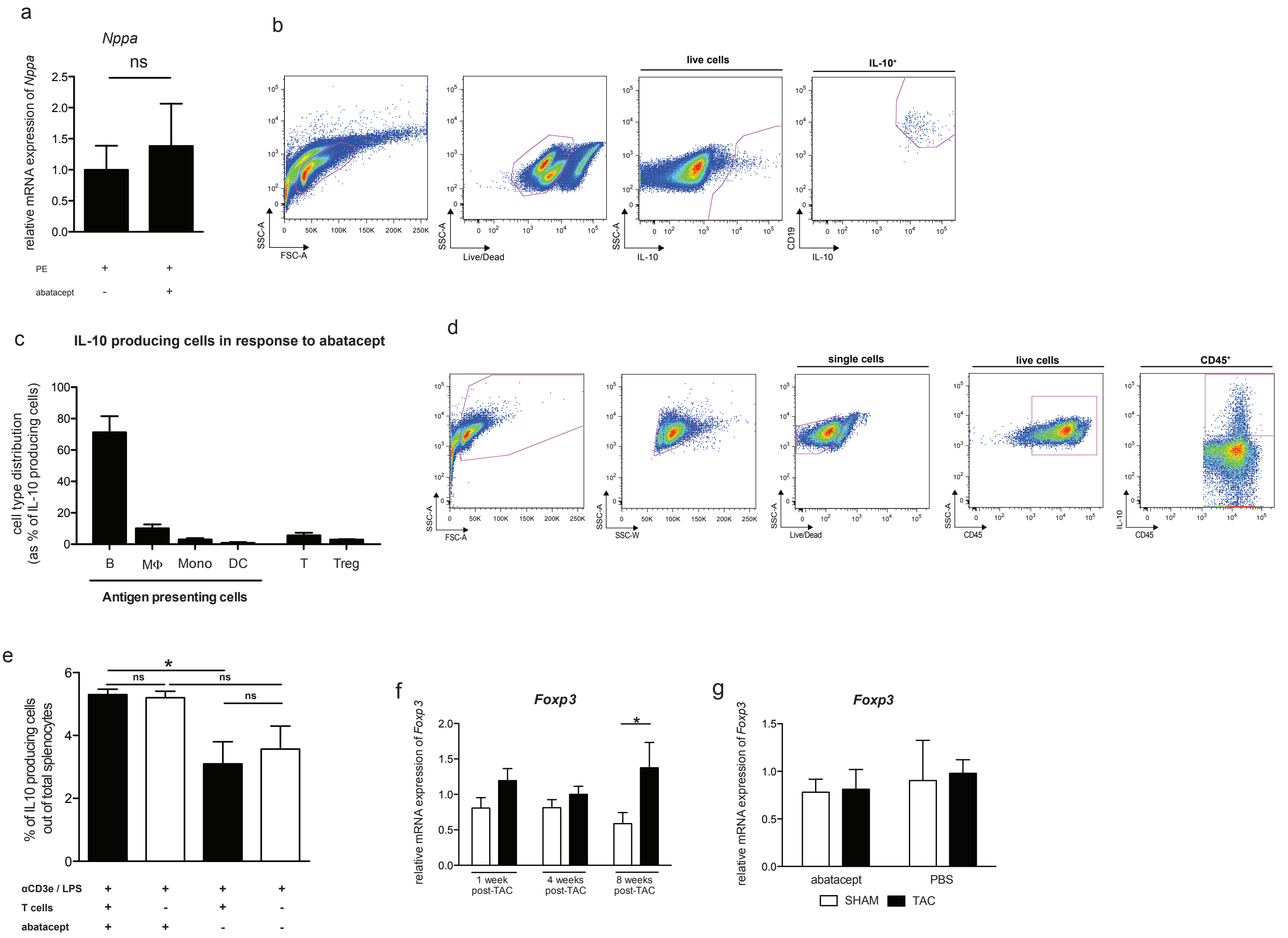

Supplementary Figure 6

(a) Evaluation of direct effects of abatacept on cardiomyocytes. Real-time qPCR of Nppa, in mouse neonatal cardiomyocytes cultured with 100 µM of phenylephrine (PE) and 20µg/ml of abatacept for 48 hours. Bars show mean ± SEM of relative mRNA expression, internally normalized to 18s rRNA (n=6). Unpaired t-test; ns, not significant. **Abatacept induces *in vitro* production of IL-10 (b)** Gating strategy for FACS analysis of IL-10 producing cells in presence of abatacept. Cells were gated based on forward and side scatter, dead cells were excluded and IL-10 producing cells were gated. Specific population frequencies were then analyzed ($CD19^+$ B cells, $CD11c^+$ dendritic cells, $CD11b^+$ monocytes and myeloid-derived cells, $F4/80^+$ macrophages, $CD3e^+$ T cells, and $CD3e^+$ $Foxp3^+$ Treg cells). **(c)** Total splenocytes of 8 week-old C57BL/6J mice activated with anti-CD3 and cultured with 20µg/ml abatacept were analyzed to identify the cellular source of IL-10 production, gated as in (b). Mean ± SEM of 3 independent experiments (n=3). **(d)** Gating strategy for FACS analysis of IL-10 producing leukocytes in response to abatacept. Cells were gated based on forward and side scatter, doublets and dead cells were excluded. $CD45^+$ cells were identified and $CD45^+$ IL-10-producing cells were assessed. **(e)** Total splenocytes or T cell-depleted splenocytes of 8 week-old C57BL/6J mice were activated with 2µg/ml anti-CD3 and 5 µg/ml of LPS. After 48 hours in culture with or without 20µg/ml abatacept were analyzed by flow cytometry for IL-10 production. Bars show mean ± SEM of 3 independent experiments (n=3) of the percentage of IL10 producing cells out of total splenocytes. Two-way ANOVA with Bonferroni post-test; *, p-value <0.05. **Presence of regulatory T cells in TAC- and sham-operated mice. (f)** Real-time qPCR for *Foxp3* in the left ventricle of C57BL6/J mice, 1, 4, and 8 weeks after TAC or sham operation. Bars show mRNA expression internally normalized to 18s rRNA expression. Mean ± SEM (n=7–9). Two-way ANOVA with Bonferroni post-test; *, p-value <0.05. **(g)** Real-time qPCR for *Foxp3* for the same samples as in Fig. 4f. Bars show mRNA expression internally normalized to 18s rRNA expression. Mean ± SEM (n=7–9). Two-way ANOVA with Bonferroni post-test; ns, not significant.

	SHAM				*TAC*			
	**Basal (n=3)**	**1 week post-sham (n=3)**	**3 weeks post-sham (n=3)**	**4 weeks post-sham (n=3)**	**Basal (n=7)**	**1 week post-TAC (n=7)**	**3 weeks post-TAC (n=7)**	**4 weeks post-TAC (n=7)**
**Age (wks)**	8.7±0.0	9.6±0.1	11.7±0.1	12.6±0.0	8.6±0.1	9.6±0.1	11.6±0.1	12,6±0.0
**Weight(g)**	22.0±1.0	22.4±0.8	23.6±0.9	23.9±1.1	22.8±1.6	22.3±1.3	24.0±1.5	24.6±1.5
**HR M - mode (bpm)**	545.7÷71.1	507.7÷93.9	549.7±66.0	598.0±29.0	577.0±66.4	530.1±48.7	517.9±87.1	606.0±36.6
**% FS**	43.4±3.1	43.3±1.4	42.9±3.0	40.3±1.7	40.5±2.4	37.7±1.2 §‡	33.2±1.5 ¤§	30.6±2.2 ¤§
**% EF**	75.7±3.5	75.6±1.5	75.0±3.5	72.1±2.1	72.3±3.6	69.0±1.6 §‡	62.7±2.1 ¤§	58.8±3.5 ¤§
**LVIDd (mm)**	3.4±0.1	3.4±0.1	3.5±0.1	3.4±0.0	3.4±0.1	3.4±0.2	3.7±0.1 ❋✧	3.7±0.3 ‡
**LVIDs (mm)**	1.9±0.1	1.9±0.1	2.0±0.2	2.0±0.1	2.0±0.2	2.1±0.1 ❋	2.5±0.1 §¤	2.6±0.3 ❋¤
**IVSd (mm)**	0.7 ± 0.0	0.8 ± 0.1	0.7±0.1	0.8±0.1	0.7±0.0	0.9±0.1 ❋¤	0.9±0.1 ❋¤	1.0±0.1 #¤
**IVSs (mm)**	1.1±0.1	1.2±0.0	1.2±0.1	1.2±0.1	1.2±0.1	1.3±0.1 ‡	1.3±0.1 ❋✧	1.4±0.1 ❋✧
**LVPWd (mm)**	0.7±0.1	0.8±0.0	0.8±0.1	0.8±0.1	0.8±0.1	0.9±0.1	0.9±0.1 #¤	0.9±0.1 ‡
**LVPWs (mm)**	1.2÷0.1	1.3÷0.0	1.2±0.1	1.2±0.1	1.2±0.1	1.3±0.1	1.3±0.1 ❋✧	1.3±0.1 ‡

‡	$p<0.05$	TAC versus sham at the same time point
❋	$p<0.01$	TAC versus sham at the same time point
✧	$p<0.001$	TAC versus sham at the same time point
¤	$p<0.05$	TAC basal versus TAC at each time point
§	$p<0.01$	TAC basal versus TAC at each time point
#	$p<0.001$	TAC basal versus TAC at each time point

Supplementary Table 1: Hemodynamic parameters for TAC- or sham-operated mice at 1, 3 and 4 weeks post-operation

	Sham								*TAC*							
	Abatacept				*PBS*				*Abatacept*				*PBS*			
	Basal (n=8)	1 week post-sham (n=7)	3 weeks post-sham (n=8)	4 weeks post-sham (n=8)	Basal (n=8)	1 week post-sham (n=5)	3 weeks post-sham (n=7)	4 weeks post-sham (n=7)	Basal (n=9)	1 week post-TAC (n=8)	3 weeks post-TAC (n=7)	4 weeks post-TAC (n=7)	Basal (n=10)	1 week post-TAC (n=10)	3 weeks post-TAC (n=10)	4 weeks post-TAC (n=10)
**Age (wks)**	9.0±0.3	9.9±0.0	12.0±0.0	12.9±0.0	9±0.3	10.1±0.0	12.0±0.1	13.0±0.0	8.9±0.0	9.9±0.0	11.9±0.0	12.9±0.0	9.0±0.3	10.1±0.0	12.1±0.0	12.9±0.0
**Weight (g)**	22.6±1.4	22.1±1.3	23.5±1.5	23.7±1.7	22.6±1.4	21.7±1.4	23.11±2.1	24.0±1.9	22.5±1.6	22.1±1.4	23.4±1.3	24±1.8	22.6±1.4	21.6±1.8	23.6±1.6	24.2±1.4
**HR M-mode (bpm)**	564.4±75.8	514.0÷56.4	602.8±68.6	614.4±44.5	564.4÷75.8	579.0±18.9	577.3±69.9	575.2±85.6	556.9÷86.1	546.6÷55.8	597.9±94.6	561.4±66.0	564.4÷75.8	544.7÷46.2	575.2±85.6	558.3±60.9
**%FS**	41.4±3.8	43.0±4.1	42.6±1.5	40.7±2.0	41.4±3.8	43.3±2.5	40.6±2.5	41.4±1.7	42.0±1.5	41.3±3.2 ✱	37.3±1.6 ✱¤	36.5±3.7 ✱	41.4±3.8	34.9±1.3 ✧	30.7±3.0 ✧	27.5±3.8 ✧
**%EF**	73.2±4.8	75.2±4.8	74.9±1.6	72.8±2.4	73.2±4.8	75.8±2.6	72.7±3	73.8±1.9	74.1±1.8	73.4±3.9 §	68.3±2.2 §#	67.2±4.8 ✱	73.2±4.8	65.2±1.8 ✧	58.9±4.6 ✧	53.9±6.2 ✧
**LVIDd (mm)**	3.4±0.1	3.3±0.2	3.3±0.2	3.2±0.2	3.4±0.1	3.1±0.2	3.2±0.1	3.1±0.3	3.4±0.1	3.3±0.2	3.5±0.2 ‡◎	3.5±0.1 ✱	3.4±0.1	3.5±0.2 ✚	3.8±0.3 ✧	3.8±0.2 ✧
**LVIDs (mm)**	2±0.2	1.9±0.2	1.9±0.1	1.9±0.1	2.0±0.2	1.8±0.1	1.9±0.1	1.8±0.2	1.9±0.1	1.9±0.2 ✱	2.2±0.2 ✱¤	2.2±0.2 ✱◎	2.0±0.2	2.3±0.1 ✧	2.6±0.3 ✧	2.8±0.3 ✧
**IVSd (mm)**	0.8±0.0	0.7±0.1	0.8±0.0	0.8±0.0	0.8±0.0	0.8±0.1	0.8±0.0	0.8±0.0	0.8±0.1	0.9±0.1 #	0.9±0.1 ◎	1.0±0.0 #	0.8 ± 0.0	0.9 ± 0.1	0.9±0.1 ✚	1.0±0.1 ✧
**IVSs (mm)**	1.2±0.1	1.2±0.0	1.3±0.0	1.2±0.0	1.2±0.1	1.3±0.1	1.2±0.1	1.2±0.1	1.2±0.1	1.3±0.0 #	1.3±0.1	1.4±0.0 #	1.2±0.1	1.3±0.1	1.4±0.1 ▲	1.3±0.1
**LVPWd (mm)**	0.8±0.1	0.8±0.1	0.8±0.1	0.8±0.0	0.8±0.1	0.8±0.0	0.8±0.1	0.9±0.1	0.7±0.1	0.9±0.1 #	1±0.1 #	0.9±0.0 #	0.8±0.1	0.9±0.1 ✧	1.0±0.1 ✧	1.0±0.1 ✧
**LVPWs (mm)**	1.2±0.0	1.3÷0.0	1.2±0.1	1.2±0.1	1.2±0.0	1.2±0.0	1.2±0.0	1.2±0.0	1.2÷0.1	1.4÷0.1 #	1.4±0.1 #	1.4±0.0 #	1.2÷0.0	1.4÷0.1 ✧	1.4±0.0 ✧	1.4±0.1 ✧

‡ $p<0.05$ TAC abatacept versus TAC PBS
§ $p<0.01$ TAC abatacept versus TAC PBS
✱ $p<0.001$ TAC abatacept veruss TAC PBS
◎ $p<0.05$ TAC abatacept verus sham abatacept
¤ $p<0.01$ TAC abatacept versus sham abatacept
# $p<0.001$ TAC abatacept versus sham abatacept
▲ $p<0.05$ TAC PBS verus sham PBS
✚ $p<0.01$ TAC PBS verus sham PBS
✧ $p<0.001$ TAC PBS verus sham PBS

Supplementary Table 2: Hemodynamic parameters for TAC- or sham-operated mice treated with abatacept or PBS, at 1, 3 and 4 weeks post-operation

Gene	Forward	Reverse
**β-Myosin** *(Mhy7)*	5'-CGCATCAAGGAGCTCACC-3'	5'-CTGCAGCCGCAGTAGGTT-3'
**Brain Natriuretic Peptide (***Nppb* **)**	5'-GTCAGTCGTTTGGGCTGTAAC-3'	5'-AGACCCAGGCAGAGTCAGAA-3'
**Atrial Natriuretic Peptide (***Nppa* **)**	5'-CACAGATCTGATGGATTTCAAGA-3'	5'-CCTCATCTTCTACCGGCATC-3'
**18S Ribosomal RNA (18S)**	5'- AAATCAGTTATGGTTCCTTTGGTC -3'	5'- GCTCTAGAATTACCACAGTTATCCAA -3'

Supplementary Table 3 : List of primers used.

-
